# Multifractal and Entropy-Based Analysis of Delta Band Neural Activity Reveals Altered Functional Connectivity Dynamics in Schizophrenia

**DOI:** 10.3389/fnsys.2020.00049

**Published:** 2020-07-24

**Authors:** Frigyes Samuel Racz, Orestis Stylianou, Peter Mukli, Andras Eke

**Affiliations:** Department of Physiology, Semmelweis University, Budapest, Hungary

**Keywords:** dynamic functional connectivity, multifractal analysis, information-theoretical entropy, machine learning, schizophrenia, electroencephalography

## Abstract

Dynamic functional connectivity (DFC) was established in the past decade as a potent approach to reveal non-trivial, time-varying properties of neural interactions – such as their multifractality or information content –, that otherwise remain hidden from conventional static methods. Several neuropsychiatric disorders were shown to be associated with altered DFC, with schizophrenia (SZ) being one of the most intensely studied among such conditions. Here we analyzed resting-state electroencephalography recordings of 14 SZ patients and 14 age- and gender-matched healthy controls (HC). We reconstructed dynamic functional networks from delta band (0.5–4 Hz) neural activity and captured their spatiotemporal dynamics in various global network topological measures. The acquired network measure time series were made subject to dynamic analyses including multifractal analysis and entropy estimation. Besides group-level comparisons, we built a classifier to explore the potential of DFC features in classifying individual cases. We found stronger delta-band connectivity, as well as increased variance of DFC in SZ patients. Surrogate data testing verified the true multifractal nature of DFC in SZ, with patients expressing stronger long-range autocorrelation and degree of multifractality when compared to controls. Entropy analysis indicated reduced temporal complexity of DFC in SZ. When using these indices as features, an overall cross-validation accuracy surpassing 89% could be achieved in classifying individual cases. Our results imply that dynamic features of DFC such as its multifractal properties and entropy are potent markers of altered neural dynamics in SZ and carry significant potential not only in better understanding its pathophysiology but also in improving its diagnosis. The proposed framework is readily applicable for neuropsychiatric disorders other than schizophrenia.

## Introduction

Schizophrenia (SZ) is a severe psychiatric disorder that can be characterized by altered perception and sensory processing, distorted thinking and impaired affective, social and cognitive functions (Uhlhaas and Singer, 2010). Yet being one of the most prevalent mental diseases affecting approximately 1% of the worldwide population (Bhugra, 2005), still no objective diagnostic test exists for SZ (Boutros et al., 2008; Calhoun et al., 2008). Moreover, the etiology of SZ is still unclear, despite being the subject of intense research for more than 100 years (Uhlhaas and Singer, 2010; Yu et al., 2015). Evidently, there has been a growing interest recently in developing tools that can yield quantitative markers of SZ with a biological basis. The expected benefits of these would be twofold: (i) advancing diagnosis and screening of the disease, while also (ii) providing further insights on its underlying neural mechanisms. The hypothesis of abnormal or altered connectivity has been suggested as a key feature of SZ (Friston and Frith, 1995; Bullmore et al., 1997), referring to it as a dysconnectivity syndrome (Friston et al., 2016). Accordingly, many recent studies utilized tools of functional neuroimaging and connectivity analyses to identify biomarkers of SZ (Arbabshirani et al., 2013; Du et al., 2015, 2018; Rashid et al., 2016).

Many SZ-related alterations of functional connectivity (FC) were revealed both at rest and during task modulation (Calhoun et al., 2009; van den Heuvel and Fornito, 2014; Kambeitz et al., 2016; Sheffield and Barch, 2016), however results from different studies are often inconsistent (Fox and Greicius, 2010). FC is most commonly defined as the statistical interdependence of neural activity recorded from disparate brain regions (Friston et al., 1993). This dependence can be captured in many ways from bivariate methods (Sakkalis, 2011; Smitha et al., 2017) to data-driven multivariate approaches such as independent component analysis (ICA) (Li et al., 2009). The large variety of available analytical tools can be considered as one of the (many possible) reasons of contradictory results (Maran et al., 2016). Recently, it has also been proposed (Damaraju et al., 2014) that the inconsistency may arise from the fact that most previous studies analyzed FC in a static manner, i.e., implicitly regarding functional connectivity constant during the measurement period (static functional connectivity, SFC). On the other hand, it has been shown that FC fluctuates even in the resting state (Chang and Glover, 2010; Hutchison et al., 2013; Allen et al., 2014). Indeed, several studies revealed alterations of dynamic functional connectivity (DFC) in SZ that could not be captured by simple SFC analyses (Damaraju et al., 2014; Ma et al., 2014).

Much progress has been made in the past decade in terms of developing methods to capture and characterize dynamic features of FC (see Preti et al., 2017 for a recent review). Among others, dynamic graph theoretical analysis has emerged as a frequently used approach (Dimitriadis et al., 2010; Tagliazucchi et al., 2012; Yu et al., 2015). Graph theory is a popular and powerful tool of FC studies (Bullmore and Sporns, 2009) and is used to describe various topological aspects of complex brain networks reconstructed from physiological data through a set of relatively simple graph theoretical measures (Rubinov and Sporns, 2010). It was also adapted to the DFC framework by multiple studies to capture the spatio-temporal evolution of functional networks (Dimitriadis et al., 2010; Tagliazucchi et al., 2012). As details of brain graph reconstruction fundamentally depend on the particular neuroimaging modality in use, functional magnetic resonance imaging (fMRI) is currently the most frequently used imaging technique. Electroencephalography (EEG) on the other hand provides a reasonable alternative with – albeit lower spatial, but – much higher temporal resolution, thus allowing for a more detailed reconstruction of network dynamics. Despite this and other advantages of EEG imaging (i.e., its accessibility and mobility), up to date not many studies have used dynamic graph analysis of electrophysiological recordings to investigate DFC in SZ (Dimitriadis, 2019).

Dynamic graph theoretical measures were reported to express reduced variance in schizophrenic patients when compared to healthy individuals (Yu et al., 2015) and features extracted by dynamic graph analysis lead to a better classification of SZ patients than simple static network measures (Lombardi et al., 2019). However, it has been shown that global FC fluctuates according to scale-free (or *fractal*) dynamics (Stam and de Bruin, 2004; Van de Ville et al., 2010). Statistical properties (such as the variance) of scale-free processes do not have a characteristic time scale, but they depend on the scale of observation according to a power-law function, and the relationship is established via the scaling exponent (Eke et al., 2000). The scale-free property manifests itself in the time domain as long-range autocorrelation, meaning that such processes have an autocorrelation function that decays according to a power-law rather than an exponential function like of those having characteristic time scales (Eke et al., 2000). Furthermore, in our recent works we showed that functional brain networks express not only scale-free/fractal but indeed multifractal dynamics (Racz et al., 2018a, b), meaning that the local scaling exponent also changes with time. More generally, mono- and multifractality has been recognized previously as a fundamental property of not only DFC but brain dynamics in general, across species and modalities (Herman et al., 2011; Nagy et al., 2017). Such dynamic features cannot be captured by simple first and second-order statistics, thus multifractal time series analysis called for providing a more detailed characterization of network dynamics. Temporal complexity of brain network dynamics can also be efficiently captured in entropy-related measures – which capture the information production rate of processes – such as sample entropy (SE) (Richman and Moorman, 2000) or permutation entropy (PE) (Bandt and Pompe, 2002). Indeed, temporal complexity of DFC has been shown to express characteristic regional patterns that reflect well the underlying functional organization of the brain (Racz et al., 2019). Similar studies revealed that patients with SZ express higher SE in their FC dynamics than healthy control (HC) individuals (Jia et al., 2017; Jia and Gu, 2019). Since the aforementioned methods appear promising tools in characterizing DFC, our main goal in this study was to investigate network dynamics in SZ by means of multifractal and entropy-related analysis. To the best of our knowledge, this is the first study applying multifractal analysis to characterize network dynamics in schizophrenia.

Beyond group-level inferences, the true utility of the extracted dynamic features would lie with their ability to enhance the discrimination of individual cases. Machine learning techniques can be used to build models for classifying individual subjects as HC or SZ, however most methods do not yield any additional information on which predictors play the most important role in the classification process. One of the exceptions is the class of random forest classifiers (RFCs) which can provide measures on the importance of each individual feature (Breiman, 2001) and thus are frequently and efficiently used not only for classification but for feature selection purposes as well (Archer and Kirnes, 2008; Menze et al., 2009). Our goal in this study therefore was not only to investigate if multifractal and entropy-related properties of DFC are altered in SZ, but also to explore how these features could serve as potential markers of the disease when classifying individual cases. We analyzed resting-state EEG recordings from healthy individuals and patients with SZ, and performed dynamic graph theoretical analysis to capture brain network dynamics. Since electrophysiological abnormalities are reported most frequently and consistently in delta band (0.5–4 Hz) neural activity (Newson and Thiagarajan, 2019), in our analysis we primarily focused on this frequency range. Besides conventional first- and second-order indices (such as the mean and variance), connectivity dynamics were characterized by their multifractal and entropy-related properties, while a traditional SFC analysis was also performed as a baseline. Apart from group-level comparisons, an RFC was trained and validated using a leave-one-out scheme, and estimates on predictor importances were extracted.

## Materials and Methods

### Participants and Data Acquisition

Resting-state EEG recordings of an openly available database published previously (Olejarczyk and Jernajczyk, 2017) were analyzed. The dataset comprised EEG records of 14 SZ patients (7 females aged 28.3 ± 4.1 years and 7 males aged 27.9 ± 3.3 years) and 14 age- and gender-matched HC individuals (7 females aged 28.7 ± 3.4 years and 7 males aged 26.8 ± 2.9 years). Subjects of the SZ group were diagnosed with paranoid schizophrenia according to the International Classification of Diseases ICD-10 criteria (category F20.0) and were hospitalized at the Institute of Psychiatry and Neurology in Warsaw, Poland. Only individuals over the age of 18 were allowed to participate in the original study and subjects of the SZ group had a medication washout period of a minimum of 1 week prior to the measurement. Exclusion criteria included organic brain pathology, first episode of schizophrenia, other neurological diseases such as epilepsy, Alzheimer’s or Parkinson’s disease, or presence of any general medical condition (for further details, see Olejarczyk and Jernajczyk, 2017). All participants were informed of the measurement protocol and provided written informed consent prior to participation. The original study was approved by the Ethics Committee of the Institute of Psychiatry and Neurology in Warsaw. The data was downloaded from the repository at http://dx.doi.org/10.18150/repod.0107441.

Measurement of all participants was performed in an eyes-closed resting-state condition where EEG activity was recorded at a sampling rate of 250 Hz from 19 cortical regions (Fp1, Fp2, F7, F3, Fz, F4, F8, T3, C3, Cz, C4, T4, T5, P3, Pz, P4, T6, O1, O2) according to the standard 10–20 montage (Nuwer et al., 1998) with an additional reference electrode placed at FCz. The original datasets consisted of 15 min of raw EEG data, from which a 3 min long artifact-free segment was selected for each participant for further analysis.

### Preprocessing

Data preprocessing was carried out in a fully automatized manner using the Batch EEG Automated Preprocessing Platform (Levin et al., 2018). The data was first band-pass filtered between 0.5 and 45 Hz with additional “cleanline” filtering at 50 Hz to remove line noise. Subsequently, artifact removal was performed using the Harvard Automated Processing Pipeline for Electroencephalography (Gabard-Durnam et al., 2018), a built-in module of BEAPP for standardized artifact removal. HAPPE was set to perform the following steps: (*i*) wavelet-enhanced ICA filtering for spike artifact removal (You and Chen, 2005), (*ii*) subsequent ICA with automated component rejection using the Multiple Artifact Rejection Algorithm (Winkler et al., 2011, 2014), and (*iii*) re-referencing against the common average reference. For ICA, HAPPE used the extended Infomax algorithm as implemented in the EEGLAB software package (Delorme and Makeig, 2004). Finally, EEG data was forward-backward filtered using a 5th order zero-phase Butterworth filter with lower and upper cutoff frequencies 0.5 and 4 Hz, respectively. Data preprocessing and subsequent analysis steps were carried out using Matlab (MathWorks, Natick, MA, United States).

### Dynamic Functional Connectivity Estimation

The Synchronization likelihood (SL) method (Stam and van Dijk, 2002) was used to estimate functional connectivity between all pairs of brain regions. SL is a dynamic measure of generalized synchronization that estimates the probability of synchronization between two processes for every time point. It utilizes a temporal embedding scheme (Takens, 1981) and looks for similarities in recurrences around every time point in a “k-nearest neighbor” manner, using the L2 (Euclidean) norm. SL requires five input parameters: the embedding dimension *m*, the embedding time lag *L*, a window parameter *w*_1_ controlling for autocorrelation effects, a window parameter *w*_2_ that serves a similar purpose as the time window in a sliding window approach and a resolution parameter *p*_*ref*_. In case of data with explicit frequency limits and fixed sampling rate – such as narrow-band EEG signals –, these parameters (except for *p*_*ref*_) can be defined in a standardized manner according to simple signal processing principles (Montez et al., 2006). Accordingly, in the current analysis we had the following set of parameters: *m* = 25, *L* = 20, *w*_1_ = 960, and *w*_2_ = 1959, while we set *p*_*ref*_ to be equal to 0.05, similarly to previous studies (Stam and van Dijk, 2002; Jalili, 2016). Being a probability-type measure, SL takes on values between 0 and 1 with 0 indicating complete lack of synchronization and 1 indicating perfect synchronization.

SL *per se* estimates synchronization of two processes in a time-resolved manner (Stam and van Dijk, 2002). Therefore, computing SL between all possible pairs of channels yielded a dynamically changing synchronization matrix (i.e., a synchronization matrix for every time point) for every subject, from which the first 2^15^ consecutive matrices were made subject for further analysis. Additionally, as a reference we also computed static FC between all channels, where static SL was acquired according to Stam and van Dijk (2002) by averaging the time-resolved values of SL. This procedure yielded only one synchronization matrix for every subject. Further details on the SL method and its appropriate parameter settings are found elsewhere (Stam and van Dijk, 2002; Montez et al., 2006).

### Graph Theoretical Analysis

The synchronization matrices were first thresholded to exclude non-significant and spurious connections (Rubinov and Sporns, 2010). For this purpose, we applied the cost-thresholding scheme introduced by Achard and Bullmore (2007). In that, for every time-point the threshold was set to a value so that only a desired fraction *K* of all connections (i.e., the strongest connections) were kept in the network. This procedure yielded dynamic weighted networks with a constant number of connections, thus graph theoretical measures truly captured the reorganizations of functional network topology. The whole analysis pipeline was carried out for multiple values of *K* ranging from 0.15 to 0.5 in 0.05 increments. The lower limit of *K* was set to 0.15 as we found that cost values below that often rendered the functional networks disconnected, while the upper limit was defined according to Achard and Bullmore (2007).

Subsequently we described the global topology of functional brain networks for every time point with graph theoretical measures connectivity strength (D), global clustering coefficient (C), and global efficiency (E). Global connectivity strength was acquired as the fraction of the sum of present edge weights and the maximal possible value of overall edge weights in the network (Rubinov and Sporns, 2010). The local clustering coefficient of a particular node can be defined as the fraction of the node’s neighbors that are also neighbors of each other (Watts and Strogatz, 1998), while the global clustering coefficient, C is the average taken over all nodes in the network. Global network efficiency is defined as the average inverse shortest path length of the network taken over all pairs of nodes (Latora and Marchiori, 2001). C is a widely used measure of segregation, i.e., how much nodes of the network (regions of the brain) tend to form densely connected groups, and characterizes information processing on the local level. On the other hand, E is a measure of integration, i.e., how the brain combines specific information distributed over disparate regions and thus it represents information processing on the global level. All weighted network measures were computed using functions of the Brain Connectivity Toolbox (Rubinov and Sporns, 2010).

This analysis yielded network measure time series (NMTS) for each cost value and graph theoretical measure, a total of 28 subjects × 8 costs × 3 network measures = 672 NMTS, that were subjected to dynamic analysis. Finally, graph theoretical analyses were also performed on the static synchronization matrices as well, yielding one value of D, C, and E for every cost, per subject.

### Dynamic Features of Brain Connectivity

First, the mean and variance (μ and σ^2^, respectively) of each NMTS were calculated. We also computed the excursion from median (*EfM*) measure recently proposed by Zalesky et al. (2014) to capture the true dynamic nature of functional brain networks. This measure was suggested to capture time-varying behavior more efficiently than the variance as it takes into account both the amplitude and the duration of periods where the process deviates from its median. *EfM* was calculated with the input parameters *a* = 0.9 and *b* = 1, as suggested by previous studies (Zalesky et al., 2014; Hindriks et al., 2016). Yet *EfM* was originally proposed as a test statistic for distinguishing true FC dynamics from random statistical fluctuations of stationary FC, here we only used it as a non-linear measure on grading of “how dynamic” functional brain network topology was.

**FIGURE 1:**
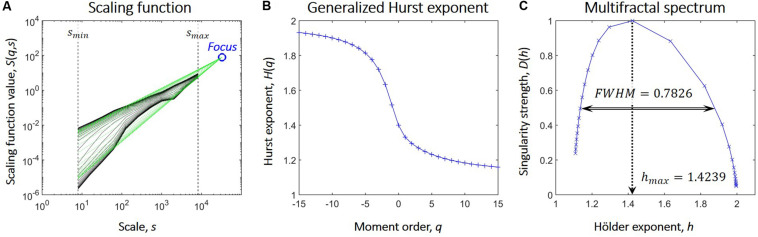
Steps of focus-based multifractal analysis. **(A)** After the scaling function (marked in black) is acquired, linear regression is used to fit power-law functions (marked in green) at each moment order *q*. On double logarithmic plots these appear as linear functions whose slopes are the scaling exponents. Also, in case of finite length signals, these converge to one point, the Focus, that is used as a reference point during regression. **(B)** The generalized Hurst exponent, *H*(*q*) is acquired as the scaling exponents of the functions fitted on the scaling function at each value of q. The focus-based formalism enforces the monotonously decreasing nature of *H*(*q*); a prerequisite for Legendre transformation. **(C)** The multifractal spectrum is acquired from *H*(*q*) via Legendre transformation and is described by the Hölder exponent at its maximal value (*h*_*max*_) and its full width at half maximum (*FWHM*).

We used the focus-based multifractal signal summation conversion (FMF-SSC) method (Mukli et al., 2015) to capture multifractal properties of the NMTS. FMF-SSC estimates the multifractal spectrum by first calculating the scaling function *S*(*q*,*s*) according to:

(1) $$S\,\left(q,s\right)={\left\{\frac{1}{N_{s}}\,\sum_{\upsilon =1}^{N_{s}}\sigma \,{\left(\upsilon ,s\right)}^{q}\right\}}^{\frac{1}{q}}$$

where s is the scale, *N*_*s*_ is the number of non-overlapping windows of size *s*, υ is the index of the window, σ(υ,*s*) is the standard deviation of the υth window at scale *s* and *q* is the moment. The generalized Hurst exponent, *H*(*q*), is then estimated by focus-based multiple linear regression for every *q* simultaneously. Finally, the multifractal spectrum is acquired via applying Legendre transformation to *H*(*q*). Consequently, FMF-SSC qualifies as an indirect approach when analyzing multifractality by providing information about the distribution of local scaling exponents of the investigated process through its multifractal spectrum. The key steps of FMF-SSC are illustrated in Figure 1, while further details of FMF-SSC and its parametrization are described elsewhere (Mukli et al., 2015). Accordingly, we performed FMF-SSC with the following settings: *s* were set according to 2^*n*^ datapoints per window with *n* ranging from 3 to 13 in steps of 1, and *q* ranging from -15 to 15 with increments of 1. The lower limit of *n* was defined to have 8 data points, while the upper limit was set to be equal to 1/4 of the signal length. FMF-SSC yields two endpoint measures, *h*_*max*_ and *FWHM*. *h*_*max*_ is the Hölder exponent at the peak of the multifractal spectrum and is strongly related (although not strictly equal) to the degree of global long-term autocorrelation of the process. *FWHM* is the full width at half maximum of the multifractal spectrum and captures the degree of multifractality, i.e., how much the local scaling exponent (and thus the local degree of autocorrelation) varies in time. Essentially, the larger *h*_*max*_ is, the stronger is the global long-term autocorrelation while the smaller *FWHM* is, the smaller is the variability of the local scaling exponent in time. A theoretical *FWHM* value of zero would mean that the scaling exponent does not change at all, and in which case the process does not express multifractality but reduces to a simple scale-free (or monofractal) process. However, even monofractal signals produce multifractal background noise when analyzed in a multifractal manner due to the finite length of real-life signals (Grech and Pamula, 2012) and the focus-based regression scheme. In order to exclude these cases, a multi-step surrogate data testing framework (Racz et al., 2019) was also carried out against 40 surrogates in each step to verify true multifractality of NMTS. By this means, we verified if time series truly expressed power-law scaling and that their *FWHM* values were significantly larger than those of strictly monofractal surrogate signals of otherwise similar properties. In all cases, NMTS were considered significantly different from their surrogates in their investigated property if it was found outside the μ±2^∗^σ range where μ and σ denotes the mean and standard deviation acquired from the surrogates. After verifying normality of surrogate indices, this yields an approximate confidence level of 0.05.

**FIGURE 2:**
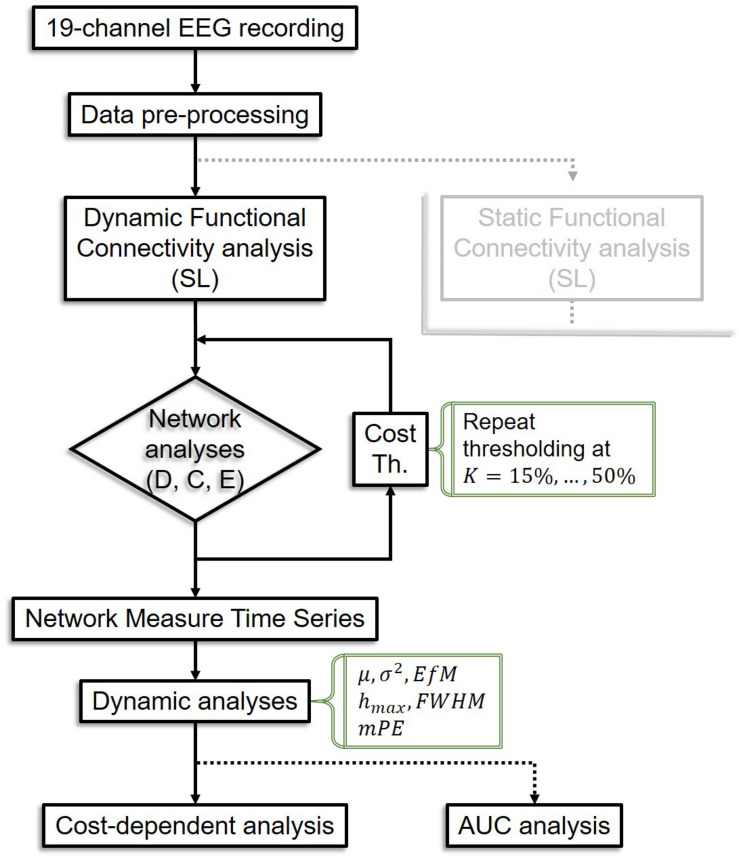
Flowchart of the analysis pipeline. The analysis pipeline for static connectivity analysis is not shown as it is equivalent in most steps to the dynamic pipeline, except that only one connectivity matrix is acquired per subject, leading to only one value for each network measure instead of a time series, thus dynamic analyses are bypassed. SL, synchronization likelihood; D, connectivity strength; C, clustering coefficient; E, global efficiency; Th, thresholding; *K*, cost; AUC, area under the curve.

Temporal complexity of NMTS was captured by their information theoretical entropy (Bandt and Pompe, 2002). Since it is possible that network topology does not change in two consecutive time points, we calculated a modified version of PE (*mPE*) that allows for this effect yet still yields accurate estimates of signal complexity (Bian et al., 2012). *mPE* also builds on the temporal embedding approach; thus its input parameters include the embedding dimension and the embedding time lag. To achieve the highest resolution possible within the current experimental setup, we set the embedding dimension to 7 and the embedding time lag to 3 according to previous studies (Staniek and Lehnertz, 2008). The analysis pipeline is summarized in Figure 2.

### Statistical Analyses

First, we compared HC and SZ groups in a cost-dependent manner. Since assumptions of a two-way repeated measures ANOVA were violated in most cases, we compared values of the HC and SZ groups for each cost separately. In case of normally distributed data and equal variances two sample *t-*tests were used, while Mann-Whitney U tests otherwise. The acquired *p*-values were corrected for multiple comparisons using the false discovery rate (FDR) approach (Benjamini and Hochberg, 1995) with level α = 0.05. Significant effect of cost on the acquired indices was verified with Friedman tests with complementary Kendall’s *W* coefficient calculation in order to estimate the concordance among subjects.

Furthermore, in order to render the results independent of cost and thus reduce dimensionality for classification (see below) we calculated the area under the curve (AUC) for all calculated network measures. AUC values of all measures in the HC and SZ groups were compared using two sample *t*-tests or Mann–Whitney *U*-tests. Note that the AUC approach is commonly used in FC studies to avoid selecting a specific cost/threshold value (He et al., 2009; Koshimori et al., 2016). However, in most DFC studies AUC values for network measures are calculated for every time point first, and then dynamics of the AUC time series are analyzed (Yu et al., 2015; Kim et al., 2017). Here we took a different approach (by analyzing dynamics first for each *K* and calculating AUC afterward), as the prior summation of values could lead to undesired effects in multifractal analysis (Nagy et al., 2017). Statistical analysis was carried out using StatSoft Statistica 13.2.

### Classification

Due to the small sample size, it is unlikely that a classifier built from this dataset would generalize well on unseen real-world data. With that in mind, our goal instead was to explore if the dynamic measures of FC described above could serve as valuable features for classifiers in the future, trained on larger datasets. Therefore, we intentionally selected a standard machine learning method where information on feature importances could be easily and readily extracted. One of such methods are random forest classifiers (RFC, Breiman, 2001). A random forest consists of a set of binary decision trees, each grown from a different bootstrap sample of the training dataset. However, unlike a regular unpruned decision tree, trees of the forest do not use all predictors but split the data using only a random subset of the features. Finally, when a new example is presented, it is subjected to all trees in the forest and the target variable is predicted by aggregating the predictions of all trees, i.e., as a “majority vote.” A big advantage of RFCs is that they provide multiple estimates on feature importances (Menze et al., 2009). From these, we selected the Gini importance, a widely used measure that captures how much prediction accuracy would be affected if the given feature was not used when splitting the data (Breiman, 2001). Although there is no theoretical limit to the number of features used for training an RFC, in most cases it is accepted as a rule of thumb that the number of features should not exceed the number of training examples. For this reason, the AUC values of seven indices (static, mean, variance, *EfM*, *h*_*max*_, *FWHM*, and *mPE*) acquired from the three network measures (D,C, and E) were used for training, resulting in a total number of 21 training features.

The sample size of the dataset did not allow for a statistically robust train-test split, so that the generalization of the model could be reliably tested. Thus, we evaluated model performance via cross-validation according to a stratified leave-one-out scheme (Calhoun et al., 2008; Rashid et al., 2016). In that, the dataset was first divided into a training and a holdout set. The holdout set always consisted of one HC and one SZ subject; thus the training set comprised the remaining 26 subjects. Then, the model was trained using data of the training set and its performance was validated on the holdout subjects. In each cross-validation run, model performance was evaluated using six standard report measures: accuracy, specificity, sensitivity, positive predictive value, negative predictive value and the AUC of the receiver-operator-characteristic (ROC) curve. Similarly, the Gini importance of each feature was extracted at the end of each cross-validation cycle. The whole process was then repeated using a different pair of HC-SZ subjects as holdout set. Each HC and SZ subject were put exactly once in the holdout set; thus the model was cross-validated 14 times. Overall classifier performance was captured in the average of the six report measures over the cross-validation runs, while the overall importance of each feature was quantified as the sum of its Gini importance over the cross-validation runs.

An RFC has many hyperparameters (parameters that have to be set before training) including but not limited to the number of trees in the forest and the allowed maximum number of features used by each tree for splitting the data. Since RFC performance can strongly depend on the appropriate setting of these hyperparameters, we performed a grid search in order to find the parameter settings that yield the best overall classifier performance. Finally, we also evaluated the performance of the classifier against surrogate datasets. In that, we carried out the cross-validation scheme described previously on 100 surrogate datasets, each acquired by randomly permuting group labels among subjects (but leaving features/predictors intact). All performance measures were compared to those of surrogate data and were considered significant if they exceeded the μ±2^∗^σ range acquired from surrogates. RFCs were implemented in Python 3.7 using the RandomForestClassifier class of the Scikit-Learn package and grid search was carried out using GridSearchCV class. Details on the hyperparameter settings of the final RFC model, as well as definitions of the performance measures are provided in Supplementary Material.

## Results

Throughout the results, for all dynamic indices the network measure it was calculated on is indicated in the left superscript, e.g., ^C^*h*_*m*a*x*_ standing for the *h*_*max*_ of clustering coefficient. AUC indices are indicated in the left subscript, e.g., ${}_{A\,U\,C}^{\text{C}}\,h_{m\,a\,x}$ refers to the AUC index calculated from the *h*_*max*_ values of clustering coefficient.

### Static Functional Connectivity

**FIGURE 3:**
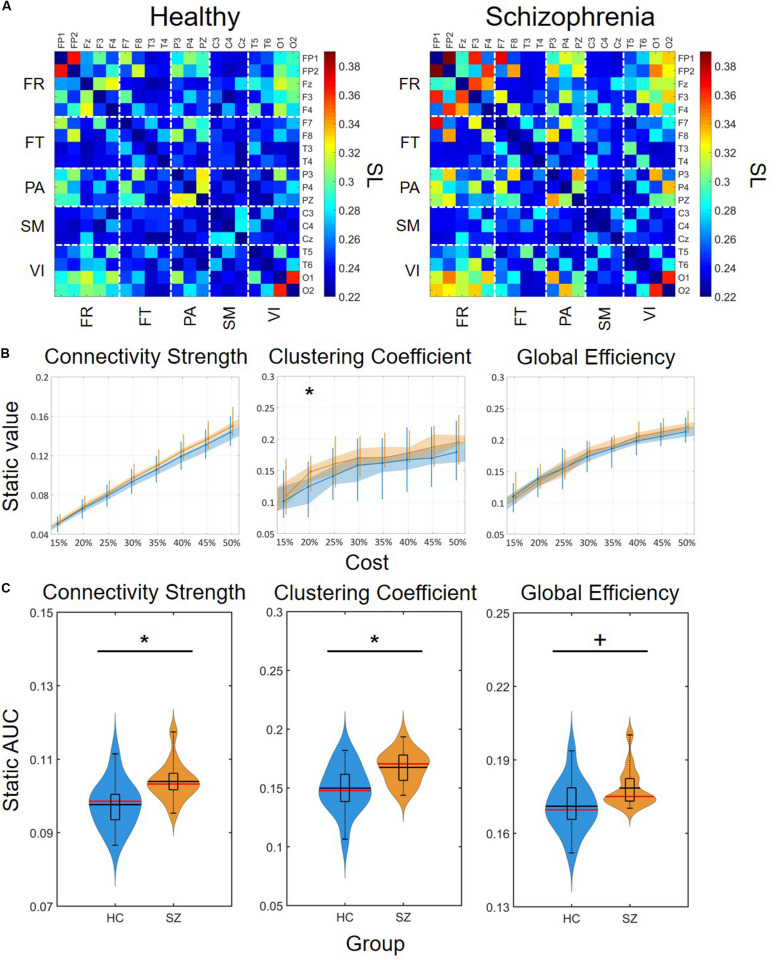
Group-average connectivity matrices and results of static functional connectivity analysis. **(A)** Group-average static connectivity matrices for healthy controls (left) and patients with schizophrenia (right). For a better comparison, the matrices are in the same color scale. Channels are grouped according to macroanatomical brain regions. **(B)** Results of the cost-dependent analysis. Data corresponding to healthy controls is marked in blue, while those of patients with schizophrenia are marked in orange. Dots mark median values, the shaded area refers to the 25th and 75th percentiles, and vertical lines range from 10th to 90th percentiles. Asterisk marks significant group difference (*p* < 0.05, corrected) acquired with two sample *t*-test. **(C)** Violin plots of static FC results for all three network measures. In each violin plot the central black line indicates the mean and the central red line indicates the median. The lower and upper horizontal lines of the rectangle mark the 25th and 75th percentile, respectively, and the outer horizontal lines indicate the 10th and 90th percentile values. The colored areas illustrate the estimated probability density function of the corresponding datasets. An asterisk marks significant group difference (*p* < 0.05) identified with two-sample *t*-test, while a plus sign marks significant difference identified with Mann-Whitney *U*-test. SL, synchronization likelihood; FR, frontal cortex; FT, frontotemporal regions; PA, parietal cortex; SM, somatomotor cortex; VI, visual cortex; AUC, area under the curve; HC, healthy control; SZ, schizophrenia.

Static synchronization matrices revealed a high degree of similarity in topology between HC and SZ groups (Figure 3A). In both groups, clusters of stronger connections were observable linking the frontal with the occipital as well as parietal regions. In these three regions, the within-regional connections also appeared to be stronger than in the rest of the network. Cost-dependent analysis showed a tendency of stronger FC in SZ for all three network measures, nevertheless, this difference was significant only in the case of C with *K* = 20% (Figure 3B). In both groups, the cost had a significant, although trivial effect on all three network measures (Table 1), as their values increased with increasing *K*. On the other hand, when we compared the AUC values acquired from D, C and E we found significant differences between the two groups, with SZ subjects expressing stronger static FC as captured in all three measures (Figure 3C).

**TABLE 1:** Effect of cost on static network measures.

		Connectivity strength		Clustering coefficient		Global efficiency	
				
		HC	SZ	HC	SZ	HC	SZ
Static	*p*	<0.0001	<0.0001	<0.0001	<0.0001	<0.0001	<0.0001
	*W*	1	1	0.8042	0.7075	1	1

*The upper row contains p-values from the Friedman tests, while the lower row contains Kendall’s coefficient of concordance (W) values. W = 1 indicates perfect agreement among subjects. HC, healthy control; SZ, schizophrenia.*

### Mean, Variance, and Excursions From Median

**FIGURE 4:**
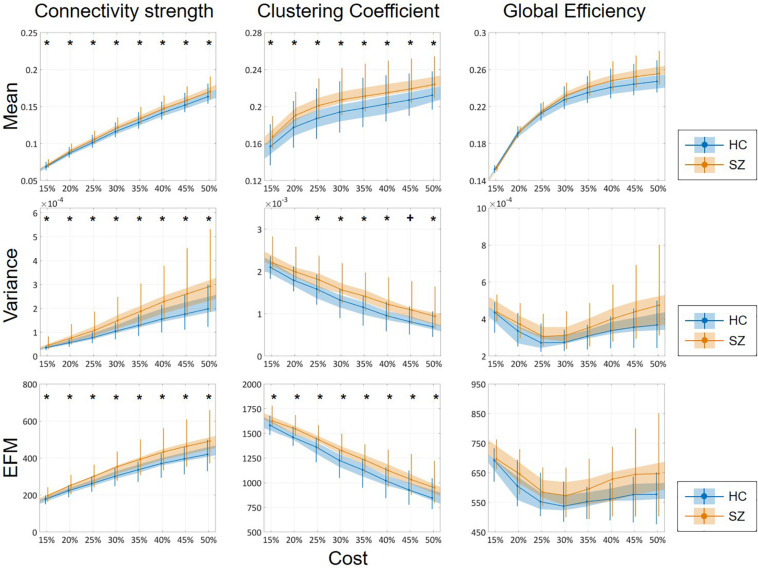
Cost-dependent results of the mean, variance and excursions from median analysis of network measures. Mean, variance, and excursions of median (EFM) values of the three network measures are plotted as functions of the cost. Black markers indicate significant group level difference (*p* < 0.05, corrected). *Two-sample *t*-test; **^+^**Mann–Whitney *U*-test; HC, healthy control; SZ, schizophrenia.

**FIGURE 5:**
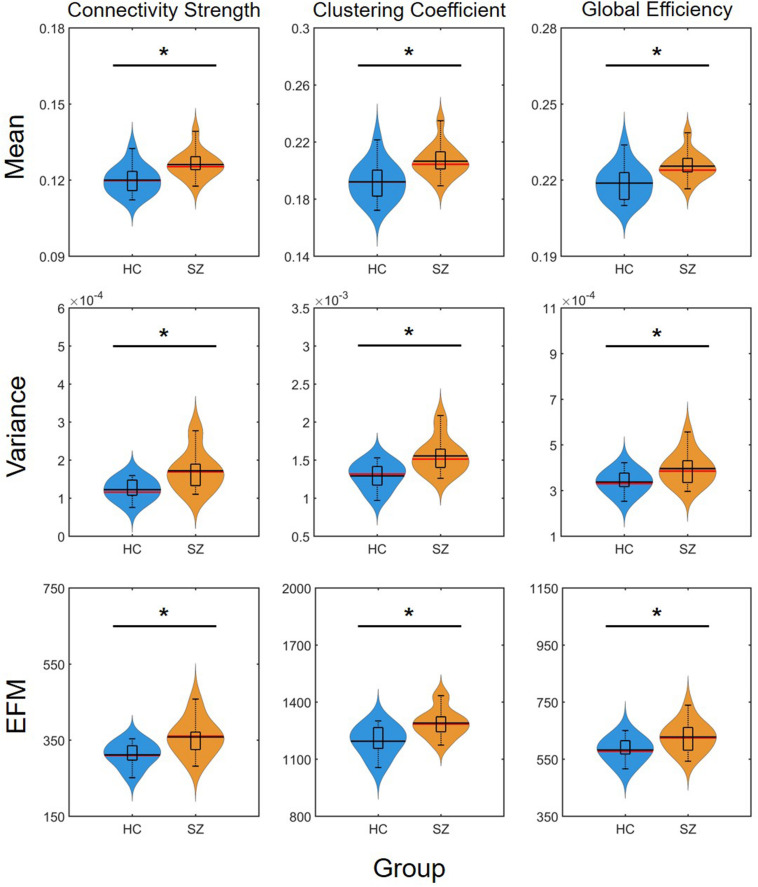
Results of the area-under-the-curve analysis regarding the mean, variance and excursions from median (EFM) of dynamic network measures. Higher mean and temporal variability of dynamic functional connectivity in SZ is apparent as captured in both connectivity strength, clustering coefficient and global efficiency. Asterisk marks significant group difference (*p* < 0.05) identified with two-sample *t*-test. HC, healthy control; SZ, schizophrenia.

The mean of DFC measures can be understood as a statistically more reliable estimation of static FC. This effect was demonstrated convincingly as the cost-dependent analysis indicated significantly higher D and C values in the SZ group with all *K* (Figure 4). As expected, cost had a similar effect on the mean of D, C and E as in the case of static FC analysis (Table 2). In addition, significantly higher variance of D and C was identified in the SZ group at almost all values of *K* (Figure 4). Interestingly, increasing the cost resulted in an increase of ^D^σ^2^ but a decrease of ^C^σ^2^, while had an indistinct effect on ^E^σ^2^. Nearly identical results to those of the variance were acquired when investigating *EfM* with additionally ^C^*E*f*M* being significantly higher in SZ for every cost value (Figure 4 and Table 2). This is in accordance with previous findings where *EfM* was found to have power equal to standard deviation in distinguishing true FC dynamics (Hindriks et al., 2016). The AUC analysis reassured stronger FC, as well as higher temporal variability of DFC in SZ (Figure 5).

**TABLE 2:** Effect of cost on the mean (μ), variance (σ^2^), and excursions from median (EfM) of dynamic network theoretical measures.

		Connectivity strength		Clustering coefficient		Global efficiency	
				
		HC	SZ	HC	SZ	HC	SZ
μ	*p*	<0.0001	<0.0001	<0.0001	<0.0001	<0.0001	<0.0001
	*W*	1	1	1	1	1	1
σ^2^	*p*	<0.0001	<0.0001	<0.0001	<0.0001	0.0001	<0.0001
	*W*	1	1	1	0.9968	0.9111	0.8365
*EfM*	*p*	< 0.0001	<0.0001	<0.0001	<0.0001	<0.0001	<0.0001
	*W*	1	1	1	1	0.7291	0.8287

*For each index, the upper rows contain p-values from Friedman tests, while the lower rows contain Kendall’s coefficient of concordance (W) values. HC: healthy control; SZ: schizophrenia.*

### Multifractal Measures and Entropy

Since multifractality can emerge due to phenomena other than the presence of long-term autocorrelations, appropriate surrogate testing is indispensable (Kantelhardt et al., 2002). In order to verify true multifractality of each NMTS, we replicated the four-step testing framework as described in detail in Racz et al. (2019). In that, we (*i*) generated surrogate time series with power-law spectra and equal spectral slope and compared goodness of fit statistics to those of the original time series, (*ii*) generated surrogates by shuffling data points of the original datasets, (*iii*) generated surrogates by phase-randomization, and (*iv*) generated strictly monofractal signals with equal global scaling exponent. In step *i* we compared the goodness of fit statistics of the spectra of the original time series to those of surrogate data with known power-law spectra, while in steps *ii*–*iv* we assessed multifractal properties of the surrogates and compared them to the original NMTS. Surrogate testing indicated that in the vast majority of cases, NMTSs expressed a power-law scaling, thus their general scale-free nature was confirmed. Shuffling reduced the process to pure white noise, as indicated by their spectral slope and *FWHM* being approximately zero. Finally, both phase randomization and monofractal signal generation produced signals with significantly smaller *FWHM* values, thus presence of true multifractality could be concluded. The percentage of NMTS that passed each test are shown for every test in Table 3. Values are reported combining both groups, as we did not find any significant difference in the fraction of NMTS that passed each test between HC and SZ groups (Mann–Whitney *U*-test, *p* > 0.05 in all cases).

**TABLE 3:** Testing results for true multifractality.

	Spectrum	Shuffling	True MF	PhaseRan
D	95.98%	100%	100%	94.64%
C	96.43%	100%	100%	100%
E	98.21%	100%	100%	100%

*MF, multifractality; PhaseRan, phase randomization; D, connectivity strength; C, clustering coefficient; E, global efficiency.*

**FIGURE 6:**
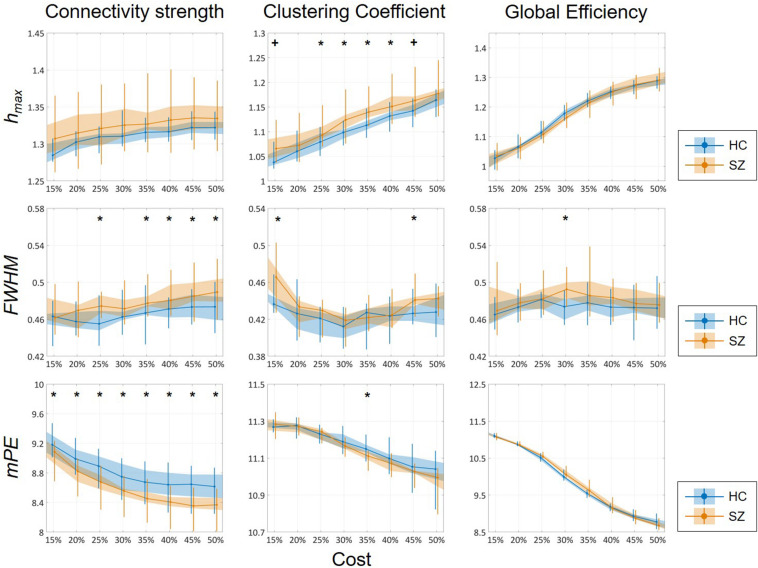
Cost-dependent results of multifractal and entropy analysis of network measures. Multifractal measures (*h*_*max*_ and *FWHM*) and modified permutation entropy (*mPE*) of all three network measures are plotted as functions of the cost. Black markers indicate significant group level difference (*p* < 0.05, corrected). *Two-sample *t*-test; **^+^**Mann–Whitney *U*-test; HC, healthy control; SZ, schizophrenia.

Cost-dependent analysis revealed significantly higher ^C^*h*_*max*_ in subjects of the SZ group for most values of *K*, while this difference appeared only as a tendency in ^D^*h*_*max*_ (Figure 6). Conversely, ^D^*FWHM* was found significantly higher in the SZ group for higher costs, while the same difference could be observed in ^C^*FWHM* and ^E^*FWHM* only at two and one cost values, respectively (Figure 6). On the other hand, ^D^*mPE* was significantly reduced in SZ subjects for all cost values, while the same difference in ^C^*mPE* was found significant only at *K* = 35% (Figure 6). Increasing *K* resulted in significant increase of *h*_*max*_ of all three network measures, while it has the opposite effect on *mPE* (Table 4). In addition, the cost had indistinct or no effect on the *FWHM* of D, C, and E.

**TABLE 4:** Effect of cost on the multifractal measures (hmax and FWHM) and modified permutation entropy (mPE) of dynamic network theoretical measures.

		Connectivity strength		Clustering coefficient		Global efficiency	
				
		HC	SZ	HC	SZ	HC	SZ
*h*_*max*_	*p*	<0.0001	<0.0001	<0.0001	<0.0001	<0.0001	<0.0001
	*W*	0.8374	0.7048	0.9788	0.9417	1	1
*FWHM*	*p*	0.0036	<0.0001	<0.0001	0.0001	0.1191	0.0069
	*W*	0.2155	0.4242	0.8861	0.3061	0.1171	0.1985
*mPE*	*p*	<0.0001	<0.0001	<0.0001	<0.0001	<0.0001	<0.0001
	*W*	<0.0001	<0.0001	<0.0001	<0.0001	<0.0001	<0.0001

*For each index, the upper rows contain p-values from Friedman tests, while the lower rows contain Kendall’s coefficient of concordance (W) values. HC, healthy control; SZ, schizophrenia.*

**FIGURE 7:**
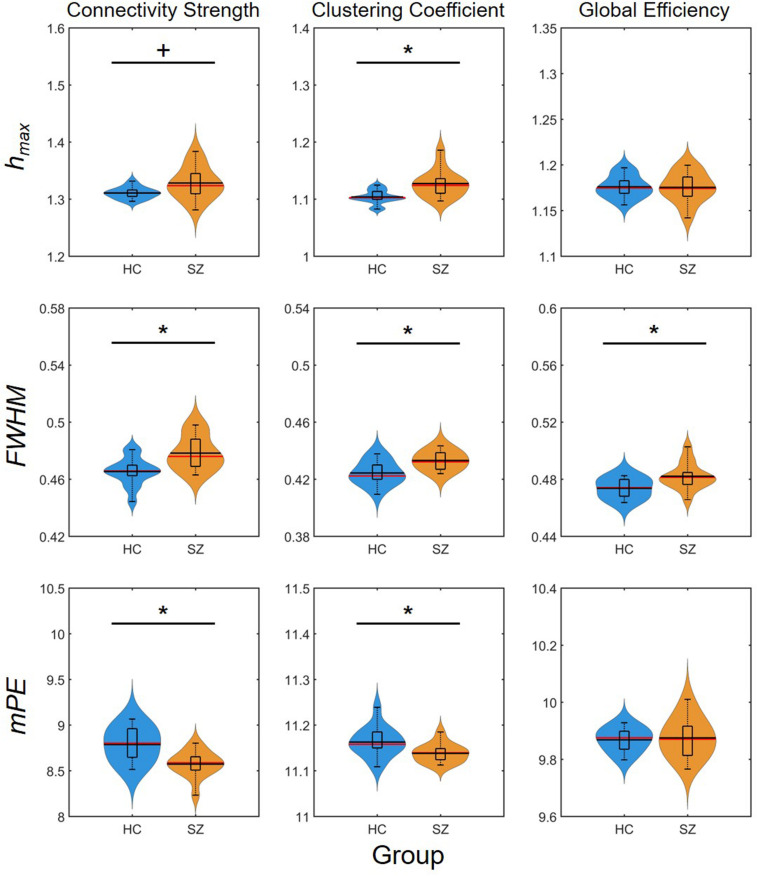
Results of the area-under-the-curve analysis regarding multifractal and entropy-related properties of dynamic network measures. Black markers indicate significant group level difference (*p* < 0.05). *Two-sample *t*-test; **^+^**Mann–Whitney *U*-test; HC, healthy control; SZ, schizophrenia; *h*_*max*_, Hölder exponent at the peak of the multifractal spectrum; *FWHM*, full width at half maximum; *mPE*, modified permutation entropy.

Again, group-level differences were found much more pronounced when comparing the AUC values of multifractal and entropy measures (Figure 7). In that, significantly higher ${}_{A\,U\,C}^{D}\,h_{m\,a\,x}$, ${}_{A\,U\,C}^{C}\,h_{m\,a\,x}$, ${}_{A\,U\,C}^{D}\,m\,P\,E$, and ${}_{A\,U\,C}^{C}\,m\,P\,E$ values were found in the SZ group, while the AUC of *FWHM* was found increased for all three network measures. This indeed highlights the power of AUC analysis as *FWHM* was found significantly higher in the SZ group only at a few cost values.

### Classification and Most Important Features

Train and test performance metrics of the classifier are shown in Table 5. Notably, the RFC was able to reach an overall 89.29% cross-validation accuracy and 100% specificity. The bottom row of Table 5 shows the mean test results for surrogate data testing with the upper boundary of the confidence interval in parentheses. Surrogate datasets yielded estimates close to chance level (50%), as expected, indicating a significantly better performance of the classifier in all metrics. The cumulative Gini importance was the highest for ^D^σ^2^, ^C^*h*_*max*_, ^C^*mPE*, and ^C^*FWHM*, highlighting the importance of dynamic indices, while in general (with the exception of *E*_*stat*_) static and mean graph theoretical measures were identified as less important for classification (Table 6). Interestingly, while ^C^*FWHM* was amongst the most important features, ^D^*FWHM* and ^E^*FWHM* were identified as negligible predictors.

**TABLE 5:** Performance report of the random forest classifier.

	Test performance					
	
	ACC (%)	SEN (%)	SPE (%)	PPV (%)	NPV (%)	ROC-AUC (%)
Train	93.41	86.83	100	100	88.55	99.32
Test	89.29	78.57	100	78.57	89.29	85.71
CI	49.93 (72.45)	46.14 (75.10)	53.71 (83.92)	35.21 (58.97)	39.00 (63.28)	51.39 (81.51)

**In the bottom row, upper boundary of the confidence interval is presented in parentheses below the mean. ACC, accuracy; SEN, sensitivity; SPE, specificity; PPV, positive predictive value; NPV, negative predictive value; ROC-AUC, area under the receiver operator characteristic curve; CI, confidence interval.**

**TABLE 6:** Feature importances extracted from the random forest classifier.

Rank	Feature	Importance
1	^D^σ^2^	3.7912
2	^C^*h*_*max*_	1.8674
3	^C^*mPE*	1.3843
4	^C^*FWHM*	1.3431
5	^D^*EfM*	1.0582
6	^E^*EfM*	1.0110
7	*E*_*stat*_	0.6700
8	^D^*h*_*max*_	0.3853
9	^E^*h*_*max*_	0.3510
10	^E^σ^2^	0.3507
11	^C^μ	0.3424
12	^D^*mPE*	0.3218
13	*C*_*stat*_	0.3104
14	^C^σ^2^	0.2683
15	^C^*EfM*	0.2224
16	^E^*mPE*	0.1630
17	^D^μ	0.1134
18	^E^μ	0.0322
19	*D*_*stat*_	0.0137
20	^D^*FWHM*	0.0
21	^E^*FWHM*	0.0

*For each index, the network measure it was calculated from is indicated in the left superscript. Static network measures are indicated by the subscript “stat” following their abbreviation. D, connectivity strength; C, clustering coefficient; E, global efficiency; stat, static; μ, mean; σ^2^, variance; EfM, excursions from median; h_*max*_, Hölder exponent at the peak of the multifractal spectrum; FWHM, full width at half maximum; mPE, modified permutation entropy.*

## Discussion

There is a growing interest in investigating dynamic features of FC in various clinical conditions (Calhoun et al., 2014; Preti et al., 2017). However, the vast majority of such studies use fMRI to capture neurodynamics, while other imaging modalities such as EEG are rather underrepresented (Mutlu et al., 2012). The orders of magnitude higher temporal resolution of EEG is a clear advantage that allows for a more detailed assessment of brain network dynamics. In this study, we reconstructed dynamic functional networks of healthy controls and patients with schizophrenia from delta-band EEG activity with a much higher sampling rate that would have been possible with fMRI. Ultimately, this allowed us to capture several aspects of temporal complexity, namely multifractality and entropy, in which our analytical framework was capable of revealing disease-related changes. In particular, DFC in SZ patients could be characterized by increased long-range autocorrelation and degree of multifractality, while lower entropy values indicated reduced temporal complexity. Furthermore, a machine learning-based classification scheme identified these dynamic connectivity features as highly relevant in classifying individual cases. Additionally, we found higher static and mean dynamic functional connectivity in schizophrenia, as well as subjects of the patient group expressed higher temporal variability in their DFC when compared to that of healthy controls.

### Aberrant Connectivity Dynamics in SZ

In the present study, we report on increased FC in SZ, as well as higher variability of dynamic graph theoretical measures in the patient group. Static approach to FC was also able to reveal this difference, although with less sensitivity than taking the means of dynamic network topological indices. In general, there is considerable inconsistency among results in the literature on resting-state dysconnectivity in SZ not only in the fMRI field (Fox and Greicius, 2010) but among electrophysiological studies, too (Maran et al., 2016). The somewhat contradictory results can be attributed to the differences in applied methods and modalities (Jalili, 2016), however independent studies using the same methodology reported both decreased (Winterer et al., 2001) and increased (Kam et al., 2013) connectivity in delta-band EEG. It also has to be noted, that the original study where the current dataset was published (Olejarczyk and Jernajczyk, 2017) performed SFC analyses using various pre-processing pipelines and FC estimators, and reported on both increased and decreased SFC in SZ, depending on the FC estimator or data pre-processing. The pre-processing pipeline in our approach was designed to be fully automatized and thus easily reproducible, however in order to investigate the plausible effects of FC estimator selection (SL in this case), we carried out the whole analysis using the exact same settings but a different, widely used estimator of connectivity, the Phase Lag Index (PLI, Stam et al., 2007). A detailed report of this analysis is provided in Supplementary Material. PLI takes a different approach from that of SL in estimating FC, as it captures synchronization of two processes based on the differences between their instantaneous phases following Hilbert transformation (Stam et al., 2007). Despite the fundamentally different nature of the two estimators, the PLI analysis yielded highly similar results (see Supplementary Material), thus making it improbable that the nature of our results was significantly biased by the choice of FC estimator.

In order to further test the robustness of the identified connectivity patterns, we also repeated the analysis pipeline using the Weighted Phase Lag Index (WPLI, Vinck et al., 2011) as the connectivity estimator. WPLI is derived from PLI by weighing the phase differences by the magnitude of the imaginary part of the cross-spectrum, and thus attributing less importance to small (i.e., close to zero) phase differences (for details see Supplementary Material), that are more susceptible to common noise sources (Vinck et al., 2011). PLI was originally introduced as an FC estimator that is robust against common source effects originating from volume conduction and/or active reference electrodes in case of EEG monitoring (Stam et al., 2007), however, WPLI was shown to further reduce these confounding factors (Vinck et al., 2011). Surprisingly, although dynamic networks reconstructed using WPLI expressed true multifractality in a proportion similarly high to those based of SL or PLI (Supplementary Table 4), between-group differences were found far less pronounced. In fact, only ^D^μ and ^E^μ indicated significantly higher connectivity in SZ. At first, this may imply that the previously observed results are not pronounced enough to be identified by more sophisticated methods such as WPLI. However, random forest classification was still able to reach comparable performance (see Supplementary Tables 8, 9), indicating that connectivity dynamics were indeed substantially different between HC and SZ individuals. The observed contrast between the results of PLI- and WPLI-based analyses may emerge from multiple origins. First, WPLI is superior to PLI when detecting phase synchronization in the presence of uncorrelated, volume-conducted noise sources (Vinck et al., 2011). Therefore, the stronger connectivity captured by PLI in SZ may arise due to the presence of more and/or stronger “noise sources” in SZ patients. Second, WPLI generally weights down phase lags, especially those close to zero. Consequently, it may be the case that most connections responsible for significant group-level differences could be characterized with small phase lags, which were effectively pruned by the WPLI calculation, thus rendering the dynamic networks indistinguishable. Although these findings highlight that one must apply great caution when interpreting the results of FC (and DFC) analyses, these issues – namely the choice of the FC estimator and specifics of the preprocessing pipeline – has also been emphasized by numerous recent studies (Jalili, 2016; Olejarczyk and Jernajczyk, 2017; Lindquist, 2020).

Previous studies applying dynamic graph analysis reported reduced mean (Du et al., 2016) and variance (Yu et al., 2015) of D, C and E in SZ patients, in contrast to our findings. Both of these studies used fMRI imaging and estimated functional network connectivity (Jafri et al., 2008) from low-frequency (0.01–0.1 Hz) spontaneous brain activity; thus a direct comparison would be difficult to make. Furthermore, the exact origins and physiological functions of wake delta-band oscillations are still debated (Dang-Vu et al., 2008; Harmony, 2013). It has been shown, that activity of resting-state networks (RSNs) reconstructed from fMRI dynamics can be attributed to not one but multiple EEG rhythms to various extents and that each RSN could be characterized with a unique set of correlations with different frequency bands (Mantini et al., 2007). For most RSNs, that largely overlap with many of the intrinsic connectivity networks (ICNs) identified by the approach of Yu et al. (2015) and Du et al. (2016), the highest correlations were found with the alpha and beta bands. Thus, it can be hypothesized that activity of these RSNs more closely resembles alpha- and/or beta- rather than delta-band activity. In order to test this hypothesis, we carried out our analysis pipeline on alpha- and beta-filtered (8–13 Hz and 13–30 Hz, respectively) EEG data as well. The analysis showed no significant differences between HC and SZ connectivity dynamics in the alpha band, while a slight (but insignificant) tendency of higher static and mean C was found in the beta band of SZ patients. On the other hand, it has been argued (Knyazev, 2012; Harmony, 2013) that waking delta activity originates not only from thalamocortical neurons (Hughes et al., 1998) but also from regions associated with the default mode network (DMN) (Raichle et al., 2001). Enhanced connectivity between thalamic and DMN regions in SZ was reported by multiple studies earlier (Skudlarski et al., 2010; Damaraju et al., 2014). In accordance with previous studies, our results of cortical delta-band dysconnectivity therefore may reflect the large-scale consequences of the involvement of these structures in SZ. Moreover, delta-band dysconnectivity in SZ also fits in with the hypothesis considering the role of wake delta rhythm in motivational, cognitive and autonomous functions (Knyazev, 2007, 2012), as these are broadly affected in SZ (Insel, 2010).

Another plausible source of the apparent contradiction between results reported in this study and those of previous works is the heterogeneous nature of SZ itself as a clinical condition (Seaton et al., 2001; Moran and Hong, 2011). It has been shown for example, that patients with various subtypes of SZ that could be characterized with largely different psychopathological symptoms expressed distinct, specific alterations in cortical electrophysiological activity (Harris et al., 2001). Likewise, several studies reported on characteristic differences in EEG findings between SZ phenotypes, i.e., those characterized mostly by positive and/or negative symptoms (Begic et al., 2000; John et al., 2009). Furthermore, brain electrical activity as assessed by EEG in SZ was shown to be affected by acute as well as chronic pharmaceutical treatment (Knott et al., 2001), the type of medication (Tislerova et al., 2008) and disease duration (Ranlund et al., 2014). These considerations, along with the drawback of no available clinical information of the subjects analyzed here, therefore prevents us to resolve this issue within the scope of this study.

### Multifractality and Temporal Complexity of DFC in SZ

One of the main contributions of this study is reporting on the true multifractal nature of DFC in SZ and its alterations compared to healthy controls. Although scale-free aspects of DFC have been known for a while (Stam and de Bruin, 2004; Van de Ville et al., 2010), its true multifractal nature was confirmed only recently (Racz et al., 2018a, b). It is a matter of debate in the neuroscience field what aspect of brain function manifests in scale-free neurodynamics (He, 2014). A view shared by many is that scale-free fluctuations are the result of an underlying self-organized critical state of the brain that gives rise for its ability to perform large-scale reorganizations quickly in response to external/internal stimuli (Linkenkaer-Hansen et al., 2001; Bullmore et al., 2009; Chialvo, 2010; Beggs and Timme, 2012; Mukli et al., 2018). In support of this hypothesis, a close correspondence was shown by Racz et al. (2018a) between dynamic graph measures (node strength in particular) and the seminal sand pile model of self-organized criticality (Bak et al., 1987). It also has been shown that self-organized critical models can express a scaling exponent different from 1 (De Los Rios and Zhang, 1999), as well as not only mono- but indeed true multifractal dynamics can emerge from systems in a critical state (Lima et al., 2017). Based on these considerations, the increased *h*_*max*_ in SZ could reflect on the impaired ability of the brain to respond to stimuli incoming from the external or internal environment. Although this hypothesis requires further research in the future, investigation of the possible correspondence between *h*_*max*_ of DFC and the severity of symptoms related to altered perception in SZ appears an important question. Note however, that criticality is by no means the only feasible explanation for the scale-free nature of brain activity. It has been argued previously, that the apparent power-law spectra of local field potential recordings could result from the extracellular medium acting as a 1/*f* filter (Bedard et al., 2006; Bedard and Destexhe, 2009). However, this mechanism alone would not explain the presence of fractal scaling in a much broader range of neural phenomena (Beggs and Timme, 2012). Simulations indicate that slow cortical oscillations may exhibit fractal scaling due to the noisy nature of dynamical synapses with sufficiently large recovery times, i.e., the combined presence of stochasticity and synaptic fatigue is required for the emergence of power-law distributions (Mejias et al., 2010). Neutral theory has been recently proposed as a plausible explanation of scale-free neural dynamics (Martinello et al., 2017), in which multiple causal avalanches can coexist (producing power-law distributions of avalanche sizes and durations) without the system being tuned or self-organized to a critical point.

True multifractality often arises from various physiological processes as the result of multiple antagonistic feedback loops (Ivanov et al., 1998; Ashkenazy et al., 2002). Feedback mechanisms play a crucial role in the generation of neural oscillations and thus synchronization (Buzsaki and Draguhn, 2004). It has been shown that by suppressing feedback regulation by administering an autonomic blockade, heart rate variability loses its multifractal nature and reduces to simple monofractal dynamics (Amaral et al., 2001). On this basis, the higher degree of multifractality of DFC could indicate stronger neural feedback regulation in SZ. Recent findings attributed increased global delta synchrony to subthreshold activity of thalamocortical GABAergic neurons (Herrera et al., 2016). As mentioned above, the exact origins of waking delta rhythm are still unknown, however, these results also point to the direction that thalamocortical neurons may play an important role (Knyazev, 2012). Furthermore, many studies support evidence for the key role of the thalamus and thalamocortical dysfunction in the pathomechanism of SZ (see e.g., Murray and Anticevic, 2017 for a review). We found increased delta connectivity as well as stronger multifractality in SZ that indeed could indicate that thalamocortical projections and feedback loops are affected, however, this hypothesis requires further research. From a more practical standpoint, multifractal dynamics often emerge from intermittent periods of larger variance due to multiplicative mechanisms (Ihlen and Vereijken, 2010), which in the terms of DFC can be understood as large-scale reorganizations of functional networks. Multiple studies argued that brain dynamics are actually more prominent during resting-state than in the presence of cognitive stimuli, as in wake rest internal thought processes and self-referential activities are unconstrained (Miall and Robertson, 2006; Deco et al., 2013). General thought processes are often distorted and disorganized in SZ patients that can be related to aberrant reorganization patterns in DFC captured as increased degree of multifractality; a plausible relationship yet to be elucidated.

Information-theoretical entropy-related measures (such as PE or SE) refer on the temporal complexity of the process with higher values implying more unpredictable behavior. Regional differences in PE has been shown to reflect the functional organization of the brain (Racz et al., 2019). It has also been reported recently that several dynamic connections of the amygdala show a decrease with aging in its complexity as measured by SE, however, this decrease was absent in patients suffering from SZ (Jia et al., 2017). Moreover, in many connections SE was higher in the HC than in the SZ group, implicating a lower dynamical complexity in the latter. Interestingly, in a subsequent study using the same dataset, the authors reported higher global SE in the SZ group that was later revealed to be the consequence of connections with higher SE in the visual recognition and auditory networks (Jia and Gu, 2019). These results may seem contradictory at first, nevertheless, in light of previous findings, they rather highlight the fact that FC dynamics vary greatly among brain regions (Racz et al., 2019) and that various regions could be affected in different ways in SZ. Our current results indicate a lower dynamical complexity of delta-band DFC in SZ. The rightful question arises of how the performance of entropy-related measures could be affected by the presence of long-range correlations. In an earlier study we found that regions with stronger autocorrelation expressed lower PE in their local FC dynamics and vice versa (Racz et al., 2019). However, according to Xiong et al. (2017) this cannot be simply a consequence of long-range autocorrelation, as it only introduces a constant bias that is independent of the degree of autocorrelation. A lower value of *mPE* implicates a lower variability in spatio-temporal patterns in a sense that the process, although varies over time, more prone to return to/repeat a specific subset of patterns instead of switching randomly between the full set. This is in line with previous DFC studies reporting that SZ patients are prone to visit fewer of the possible meta-states than HC subjects (Miller et al., 2014a, b).

It should be noted, that the obtained values for *h*_*max*_, *FWHM*, and *mPE* all indicate the presence of complex temporal structuring in connectivity dynamics. In order to emphasize this, we generated *n* = 100 random dynamic networks with equal size to those reconstructed from EEG data, in which for each time point all edges were randomly drawn from a distribution approximating that of the edges of the original networks (a normal distribution with mean 0.3 and variance 3^∗^10^–4^). The networks were thresholded at *K* = 0.35. Network measures were calculated for every time point and then multifractal and entropy analyses were carried out using the same settings as previously. As expected, all obtained indices (*h*_*max*_, *FWHM* and *mPE*) were found significantly different (*p* < 10^–8^ in all cases) from those of real networks. In fact, they were found very similar to those acquired for random noise (shuffled) time series used in surrogate data testing (*h_*max*_* = 0.513 ± 0.017; *FWHM* = 0.240 ± 0.007 and *mPE* = 12.18 ± 0.003 with *p* > 0.05 in all cases expect ^D^*mPE* of HC and ^D^*FWHM*, ^D^*mPE* and ^E^*h*_*max*_ of SZ). Notably, the same values were obtained for all three network measures. These results further emphasize that dynamic brain networks express complex temporal structuring, which is absent in dynamic networks with randomly fluctuating connection patterns.

While the SL- and PLI-based analyses led to largely similar results, some differences found regarding the cost-dependence of fractal properties and *mPE* are worth noticing (see Figure 6 and Supplementary Figure 4). Namely, increasing the cost thus including more of the weaker connections led to an increase of ^C^*h*_*max*_ and decrease of ^C^*mPE* in SL-derived dynamic networks. In contrast, the opposite pattern emerged in networks reconstructed from the PLI-based analysis. This implies that weak links in PLI networks introduce new information (that can also be understood as increased unpredictability) to network dynamics, while weaker links in SL analysis carry redundant information as their inclusion reduces dynamical complexity and increases autocorrelation. In other words, it seems as weaker links destabilize PLI but stabilize SL networks. This is indeed an interesting finding from the perspective of dynamic networks and requires further research.

### Automated Classification of Patients With SZ

One of the major critiques of the FC field is that although it was able to reveal characteristic alterations of various diseases on the group level, its actual utility in the diagnosis of individual cases is yet to be shown (Papo et al., 2014). Thus, recently more and more studies attempt to utilize SFC and DFC features to build classifiers in order to explore their true utility, especially in the diagnosis and differential diagnosis of SZ (Calhoun et al., 2008; Arbabshirani et al., 2013; Du et al., 2015; Kim et al., 2016; Rashid et al., 2016). Our model was able to reach a high cross-validation performance, comparable to those of most recent reports. Additionally, this high-level performance could be replicated when using AUC features from the PLI-based analysis (Supplementary Table 5). Note that many studies reported performance results surpassing ours, however all of these studies worked with larger sample sizes. On the other hand, a study working with the exact same dataset made available by Olejarczyk and Jernajczyk (2017) was able to reach a 71.4% accuracy and 80% balanced accuracy using an RFC model and narrow-band power values as features (Buettner et al., 2019). In a subsequent report, using data augmentation by segmenting the data sets into 1 min epochs, thus arbitrarily increasing sample size, the same group reported an outstanding 96.8% accuracy (Buettner et al., 2020). Oh et al. (2019) also used the same dataset and fit a convolutional neural network model on EEG data to classify HC and SZ subjects. They also divided the data into 25 s long epochs and were able to reach 98.1% accuracy. Although these results highlight the importance of a large sample size, the reported high accuracies may be biased, as the epochs used in the training and test sets were not independent (i.e., segments acquired from the same subject could be present both in the training and test sets). Namely, this way the classifiers could also learn and use subject-specific patterns for classification of the epochs. This is supported by the fact that when Oh and colleagues used a cross-validation scheme where data was split on a subject-based manner (i.e., epochs of each subject only appeared in either the training, validation or test sets), the accuracy of their model dropped to 81.3% (Oh et al., 2019). Considering the small size of the dataset, even though our classifiers performed reasonably well, it is unlikely that they would generalize well to real world-data. Therefore, we rather considered the RFC model as a tool for exploring which features are the most important for classification. From our results, it is apparent that static FC measures carry less, though still relevant information when compared to dynamic indices. On the other hand, ^C^*h*_*max*_, ^C^*mPE*, and ^C^*FWHM* appear promising indices of DFC besides the more commonly used σ^2^. Nevertheless, the results reported here are essentially in agreement with those of previous studies reporting on the superiority of DFC- over SFC-derived features (Rashid et al., 2016).

### Comparison With Existing Methods

In order to further clarify the advantages and plausible disadvantages of our analytical pipeline, it is indispensable to compare it with those already published in the literature. Since it could be inconclusive to draw correspondences between DFC approaches with vastly different methodologies, here we selected three previous studies utilizing dynamic graph theoretical analysis for comparison, namely those of Dimitriadis et al. (2010), Tagliazucchi et al. (2012), and Yu et al. (2015). The summary of the details is shown in Table 7. Similarly to our study, Dimitriadis et al. (2010) used EEG for monitoring brain activity, while Tagliazucchi et al. (2012) and Yu et al. (2015) estimated connectivity dynamics based on fMRI measurements. This – among other specifics such as sampling rate or temporal resolution – inherently influenced how nodes of the reconstructed networks were defined. In the EEG-based approaches nodes corresponded to recording sites (EEG channels), while Tagliazucchi et al. (2012) selected 90 cortical and subcortical regions according to the Automated Anatomical Labeling template (Tzourio-Mazoyer et al., 2002) and Yu et al. (2015) investigated connectivity between 48 ICNs (sets of brain regions forming functional units). All studies utilized a sliding window approach; however, in both EEG-based studies the window length was adaptively defined to fit the frequency characteristics of the data, while in the fMRI studies it was set according to empirical considerations. The advantages of the adaptive approach are that it reduces the number of subjective parameters of the analysis pipeline, as well as it always yields a complete characterization of the dynamics, while a short time window (e.g., 40 s) prevents slow fluctuations to fully manifest, especially if the data is filtered (e.g., between 0.01 and 0.1 Hz). Most fMRI-based DFC approaches use Pearson cross-correlation (or an inherently related similarity index such as in Yu et al., 2015) as FC estimator, that only allows for the identification of linear interactions. On the other hand, Dimitriadis et al. (2010) computed dynamic Phase-Locking Index, while in this study we used Synchronization Likelihood for DFC estimation and PLI (and WPLI) for verification. These latter measures are able to capture non-linear interactions, which is considered as an inherent feature of functional coupling between neuronal assemblies (Friston, 2000). Note, that all three studies discussed here utilized only one FC estimator and did not validate their results with a different method. All studies took different approaches for network thresholding except for that of Yu et al. (2015), where no additional threshold was applied. Dimitriadis et al. (2010) introduced a novel algorithmic technique for the objective selection of the most relevant edges, while similarly to our approach Tagliazucchi et al. (2012) used cost thresholding. However, while in the latter case the authors selected only one cost value (*K* = 0.1) here we also explored the effect of cost on network dynamics, which were revealed to be significant and characteristic to the FC estimator used, as discussed previously. All studies characterized the reconstructed networks with mostly similar network measures (see Table 7), with the larger number of nodes also allowing Tagliazucchi et al. (2012) to estimate more sophisticated network characteristics such as betweenness centrality. In this aspect our study is clearly the most constrained among those discussed here, operating on networks with the smallest number of nodes. Network size inherently limits the set of graph theoretical measures that could reasonably be used for network characterization (Rubinov and Sporns, 2010; van Wijk et al., 2010), however previous results suggest that D, C, and E could still provide valuable information even in case of small networks (Racz et al., 2017). Finally, in all studies the acquired NMTSs were analyzed in different fashions and utilized for various purposes. Dimitriadis et al. (2010) utilized the technique of replicator dynamics to identify consistent hub regions of cortical structures. Tagliazucchi et al. (2012) used correlation analysis to unfold the electrophysiological correlates of fMRI-based connectivity fluctuations. Finally, Yu et al. (2015) identified altered connectivity dynamics and patterns in SZ patients when compared to HC subjects. A common pattern of the aforementioned three studies though is that dynamic graph theoretical measures were finally reduced to their mean, while their dynamics were characterized by their variance or standard deviation (see Table 7).

**TABLE 7:** Comparison of various DFC approaches based on dynamic graph-theoretical analysis.

	Dimitriadis et al.	Tagliazucchi et al.	Yu et al.	Racz et al.
Modality	EEG	fMRI	fMRI	EEG
Node definition	Recording sites	AAL-defined brain regions	Intrinsic connectivity networks	Recording sites
Number of nodes	*N* = 30	*N* = 90	*N* = 48	*N* = 19
Window length	Adaptive to frequency range	60TRs (≈2 min)	20 TRs (40 s)	Adaptive to frequency range
Connectivity estimator	Phase-Locking Index	Pearson cross-correlation	Similarity index	Synchronization Likelihood, Phase Lag Index
Thresholding	Algorithmic identification of most significant edges	Cost thresholding at *K* = 0.1	No thresholding	Cost thresholding with *K* ranging from 0.15 to 0.5
Network measures	Global efficiency, local efficiency, small-worldness	Clustering coefficient, average path length, betweenness, small-worldness	Connectivity strength, clustering coefficient, global efficiency	Connectivity strength, clustering coefficient, global efficiency
Analysis	Mean Identification of consistent hubs by using the technique of replicator dynamics	Standard deviation Correlations of time-varying graph measures with dynamic frontal-, central- and occipital band-limited power	Variance Identification of reoccurring connectivity states	Mean, variance, *EfM* Scale-free (multifractal) analysis Temporal complexity (information content)

*EEG, electroencephalography; fMRI, functional magnetic resonance imaging; N, number of nodes; K, cost; EfM, excursions from median.*

Accordingly, one of the main contribution of our approach lies with the analysis of the multiscale and information-theoretical aspects of connectivity dynamics. Although the studies discussed above all provided valuable insights on physiological and pathological brain function, they mainly neglected the already established scale-free nature of DFC (Stam and de Bruin, 2004; Racz et al., 2018a, b). On the other hand, our approach reveals the complex temporal structuring of connectivity fluctuations that otherwise remain undetected for most approaches. The results presented here not only provide further confirmation that multifractality is an inherent property of brain dynamics, but also demonstrate that multifractal and entropy-related properties of DFC could carry significant clinical potential. In that, they could not only be utilized as disease biomarkers but may also provide further insights on the underlying mechanisms of neuropsychiatric morbidities. Note that the methodology implemented here for reconstructing time-varying brain graphs does not differ substantially from those of previous approaches. Consequently, the framework put forward in this study is readily adaptable for other DFC studies utilizing different imaging techniques or investigating neuropsychiatric disorders other than SZ.

### Limitations and Future Directions

Clearly, the most severe drawback of the present work is the lack of clinical data on SZ subjects such as illness duration, medication or positive and negative symptom scores. Although we revealed several differences between HC and SZ groups, the physiological bases of these findings remain elusive until their correspondence with clinical symptoms is investigated. Furthermore, simultaneous fMRI-EEG measurements would be also important not only for unfolding the neural basis of delta synchronization but to reconcile contradictory results within the FC field. The low spatial resolution (19 regions) is the source of yet another limitation. A replication of this study using high-density EEG (e.g., 128 or even 256 channels) would benefit from a more detailed functional network reconstruction and also allow for reliable source reconstruction with a reasonable spatial resolution (although source reconstruction can be performed using only 19 channels as well (e.g., Vecchio et al., 2020). This way plausible volume conduction effects could be further reduced and information could be gained on the involvement of specific – even subcortical – brain regions as well, thereby enhancing the interpretation of the results. A high-density setup would also allow for detailed local connectivity analyses which appear increasingly relevant in the light of recent advancements recognizing the importance of not only temporal, but spatial- and spatiotemporal patterns in DFC (Iraji et al., 2020). Specifically, it has been demonstrated by previous studies that regional alterations of DFC could play a relevant role in SZ (Damaraju et al., 2014; Jia et al., 2017; Jia and Gu, 2019), which may will be overlooked when investigating network characteristics on the global level only. Considering in addition, that multiscale and entropy-related properties of DFC were shown to express significant regional variability over the cortex (Racz et al., 2019), an extension of the current framework to the analysis of local connectivity dynamics appears as an important future research direction. In this study, only datasets of 14-14 HC individuals and SZ patients were analyzed, that limits the applicability and power of machine learning classifiers. Most importantly, using datasets of a larger sample size would allow for a train-test split scenario where the training data itself would be sufficient to perform the cross-validation and thus would allow fine-tuning of model parameters before evaluating the true model performance on previously unseen data. Small sample size also limits to some extent the possible number of features that can be used in a model. Although solutions (such as penalization in case of logistic regression or the “dropout” technique in case of neural networks) exist to circumvent this problem and prevent overfitting, in most cases it is accepted as a rule of thumb that for reliable performance the number of cases should surpass the number of features in a model (Hastie et al., 2009). Thus, increasing the sample size would also permit the inclusion of other, non-connectivity derived predictors commonly used in EEG analysis such as band-limited power. Multifractal indices appeared as important predictors, however a drawback of fractal- and especially multifractal analysis is that it requires sufficiently long (i.e., at least a few thousand data points) signals to obtain reliable estimates (Eke et al., 2000; Mukli et al., 2015). This makes multifractal analysis unfeasible for fMRI-based DFC analyses, where time series are usually in the range of hundreds of data points. Note, that PE (and *mPE*) does not suffer from this limitation (Bandt and Pompe, 2002) and is readily applicable to short time series as well. It is important to highlight that the analysis pipeline was designed deliberately to be fully automatized. This includes steps of pre-processing as well as parameter settings of the applied analysis methods that were defined based on purely data-driven considerations, thus the procedure could be easily applied to different datasets. This greatly enhances the potential of the proposed pipeline for clinical applications, as in clinical settings practicality is an important aspect. Finally, the utility of potential biomarkers lies with not only in separating healthy from patient groups but also in differentiating between diseases with similar and/or overlapping clinical manifestations, such as schizophrenia, bipolar disorder and schizoaffective diseases. Thus, further work is required to investigate disease-related alterations of the dynamic indices proposed in this study in neuropsychiatric morbidities and conditions other than schizophrenia.

## Conclusion

In summary, by applying dynamic graph theoretical analysis to EEG signals, we found delta-band dysconnectivity in patients with SZ. The SZ group expressed higher average and variance of network measures when compared to HC. Moreover, here we first report the multifractal nature of DFC in SZ that expressed stronger fractal scaling and degree of multifractality than in healthy controls. In accordance with previous studies, lower temporal complexity of DFC in SZ was captured with *mPE* analysis. Random forest classifiers indicated that indices of complexity, such as multifractality and entropy were amongst the most important predictors of the disease. This implies that these features carry great potential as biomarkers of SZ for future studies, that could facilitate its biologically- rather than symptom-based diagnosis and progression monitoring.

## Data Availability Statement

All datasets presented in this study are included in the article/Supplementary Material.

## Ethics Statement

The studies involving human participants were reviewed and approved by the Ethics Committee of the Institute of Psychiatry and Neurology in Warsaw. The patients/participants provided their written informed consent to participate in this study.

## Author Contributions

FR designed the study and the analysis framework, performed the analyses and implementation, wrote the first draft of the manuscript, and prepared all figures and tables. OS contributed to the data preprocessing and manuscript development. PM contributed to the statistical analyses, data visualization, and manuscript development. AE provided conceptual guidance during the study and contributed to the manuscript development. All authors contributed to, reviewed and gave approval on the final version of the manuscript.

## Conflict of Interest

The authors declare that the research was conducted in the absence of any commercial or financial relationships that could be construed as a potential conflict of interest.
