# Supplementary material for: Multifractal and Entropy-Based Analysis of Delta Band Neural Activity Reveals Altered Functional Connectivity Dynamics in Schizophrenia
Source: Front Syst Neurosci. 2020 Jul 24;14:49. doi: 10.3389/fnsys.2020.00049 (PMC7394222; doi:10.3389/fnsys.2020.00049)
Supplement: Supplementary file 1 [file Data_Sheet_1.pdf]

1 Phase Lag Index

The Phase Lag Index (PLI) was introduced by Stam *et al.* (2007) as a measure of functional connectivity (FC) that is robust against the influence of common sources, a problem often encountered in EEG-based FC studies due to volume conduction effects or active reference electrodes (Stam *et al.*, 2007). PLI builds on the concept of the Phase-locking value (R), a measure of phase synchronization between two dynamic processes proposed by Rosenblum *et al.* (1996). In that, two processes are considered phase-locked if the difference between their instantaneous phases remain (approximately) constant over some time period (Rosenblum *et al.*, 1996, Stam *et al.*, 2007). R takes on values between 0 and 1, with 0 indicating unsynchronized phases and one indicating perfect synchronization i.e. the phase difference remains constant throughout the investigated time period. PLI extends this concept by discarding phase differences centered around 0 mod π, that imply instantaneous influence on both processes, most likely coming from a common source (either due volume conduction or active reference electrodes). This is achieved by calculating an "asymmetry index" of the distribution of phase differences, since an asymmetric distribution centered around 0 would imply phase locking with a non-zero phase difference, while in case of independent processes this distribution is assumed to be flat and symmetric (Stam *et al.*, 2007). More precisely, if $\Delta\varphi(t)$ denotes the time-resolved differences between the instantaneous phases of two processes, PLI can be calculated according to Stam *et al.* (2007) as

$$PLI = \left|\langle sign\big(\Delta\varphi(t)\big)\rangle\right|, \qquad (1)$$

where $|\cdot|$ denotes the absolute value, $\langle\cdot\rangle$ denotes the arithmetic mean and $sign(\cdot)$ is the sign function. Similarly to R, PLI also ranges between 0 and 1, where 0 indicates either no coupling or a locked phase difference centered around 0 mod π (thus excluding instantaneous effects), while 1 indicates perfect phase-locking at a constant phase difference different from 0 mod π. For further details on phase synchronization and PLI the reader is referred to the original works of Rosenblum *et al.* (1996) and Stam *et al.* (2007), respectively.

2 Weighted Phase Lag Index

The Weighted Phase Lag Index (WPLI) is an extension of PLI that further reduces the effect of volume conducted common sources (Vinck *et al.*, 2011). This is achieved by weighing each phase difference by the magnitude of the lag, thus phase differences near zero that are more likely to represent common sources and/or more susceptible to the presence of random noise contribute only marginally to the calculation of WPLI (Vinck *et al.*, 2011, Hardmeier *et al.*, 2014). Accordingly, WPLI can be obtained as

$$WPLI = \frac{\left|\langle|\Delta\varphi(t)|\rangle sign\big(\Delta\varphi(t)\big)\right|}{\langle|\Delta\varphi(t)|\rangle}. \qquad (2)$$

WPLI is also bounded between 0 and 1, with 0 indicating uncoupled dynamics while 1 indicating perfect phase synchronization. Note however, that WPLI attributes the most weights to phase differences at 90°.

3 Dynamic functional connectivity analysis using PLI

Unlike Synchronization Likelihood (SL), PLI *per se* is not computed in a dynamic manner, thus we adapted this measure to a sliding window approach. To remain consistent in our analyses, we defined the window length as $2 * (w_1 + w_2) = 2 * (960 + 1959)$ data points (thus using the same amount of data for FC estimation at every time point as in the case of SL) and used a step size of one data point (Stam and van Dijk, 2002). We calculated PLI between all possible pairs of channels for every time point, yielding a dynamic connectivity matrix i.e. one matrix for every time point per subject. Subsequently, for every subject the first 2^{15} consecutive matrices were made subject for further analysis. For the static FC estimation, we calculated PLI in a pairwise manner using the first 2^{15} data points of the preprocessed EEG datasets, yielding one connectivity matrix. All following steps of the analysis – namely cost-thresholding, network measure estimation, dynamic analyses (including multifractal analysis and modified permutation entropy estimation) and statistical analysis – were carried out with the exact same settings as described in the main text. Note, that by applying cost-thresholding instead of weight-thresholding (where first a fixed threshold weight is set, and then all connections below that are discarded) both SL- and PLI- derived networks had the same connection densities and thus reorganizations of network topology took place roughly on the same scale. This is important, as though both SL and PLI are bounded between 0 and 1, they took on their values at rather different ranges with PLI and SL mostly being bounded in the range [0; 0.1] and [0; 0.4], respectively (see **Figure 3A** and **Figure S1A**). Finally, machine learning classification was again carried out following the exact same pipeline as described in the main text.

4 Dynamic functional connectivity analysis using WPLI

The WPLI-based connectivity analysis was carried out in the exact same fashion as described previously for PLI.

5 Results derived from PLI- and WPLI based analyses

Results are presented in the same manner as those in the main text, using the same notation. While results obtained from PLI-based analysis are discussed in the same detail as those in the main text, only findings that indicated significant between-group differences are included from the WPLI-based analysis (except for summary statistics of the surrogate data testing for true multifractality, which is also reported here).

5.1 Static functional connectivity

Static synchronization matrices again revealed a high degree of similarity in topology between HC and SZ groups (**Supplementary Figure 1A**). The clusters of stronger connections linking the frontal with the occipital as well as parietal regions were found less defined in comparison to SL matrices. Moreover, in contrast to results acquired from SL analysis, neither cost-dependent (**Supplementary Figure 1B**) nor AUC analysis (**Supplementary Figure 1C**) revealed any significant differences between HC and SZ groups, although a tendency of higher D, C and E in SZ was still apparent. As expected, the cost had a significant effect on all three network measures in both groups (**Supplementary Table 1**), with their values increasing as a function of *K*.

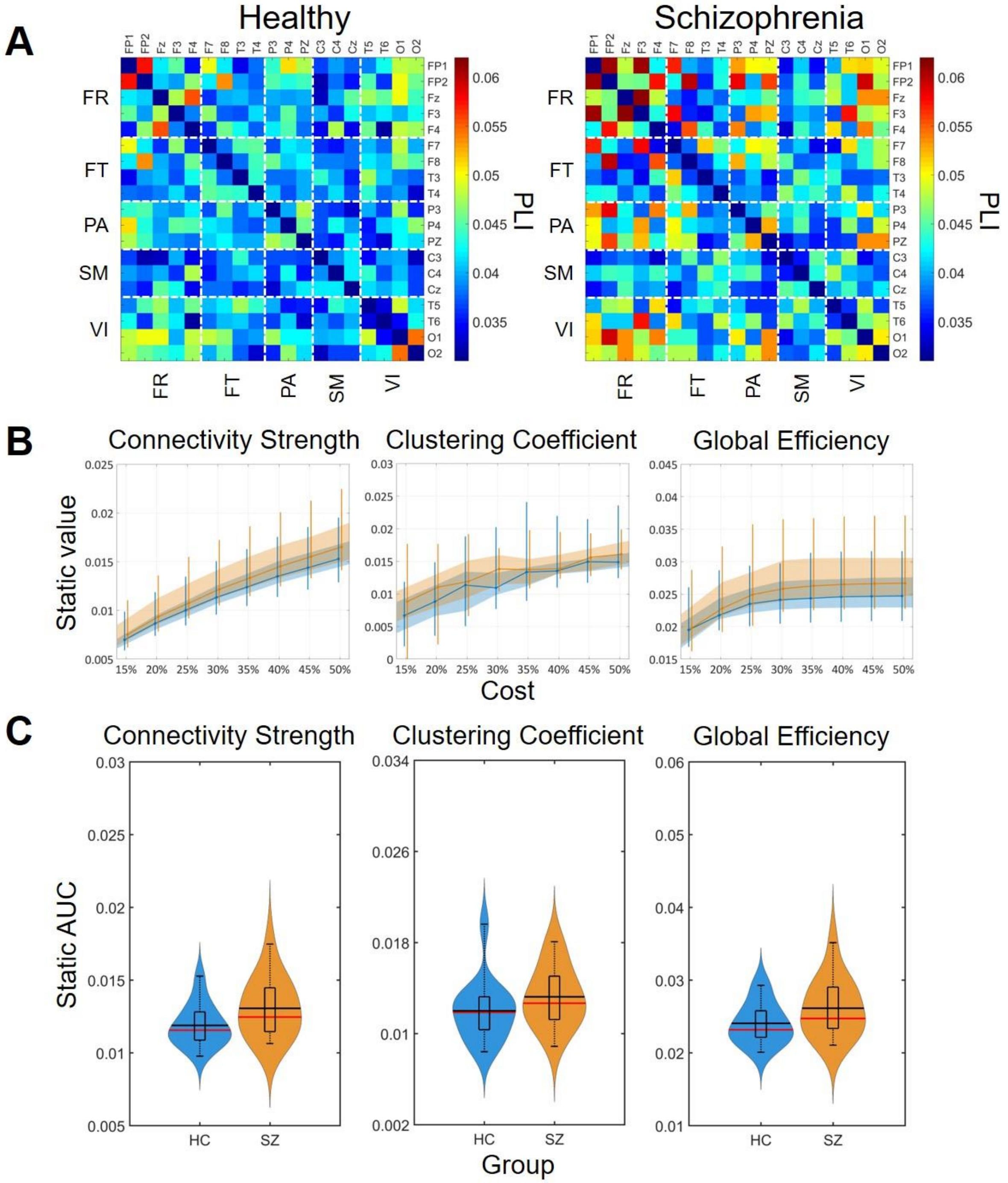

Supplementary Figure 1. *Group-average connectivity matrices and results of static functional connectivity analysis.* A: Group-average static connectivity matrices for healthy controls (left) and patients with schizophrenia (right). Channels are grouped according to macroanatomical brain regions. B: Results of the cost-dependent analysis. Data corresponding to healthy controls is marked in blue, while those of patients with schizophrenia are marked in orange. Dots mark median values, the shaded area refers to the 25th and 75th percentiles, and vertical lines range from 10th to 90th percentiles. Asterisk marks significant group difference (p<0.05) after false discovery rate acquired with two sample t-test. C: Static FC results for all three network measures. In each violin plot the central black line indicates the mean and the central red line indicates the median. The lower and upper horizontal

lines of the rectangle mark the 25th and 75th percentile, respectively, and the outer horizontal lines indicate the 10th and 90th percentile values. The colored areas illustrate the estimated probability density function of the corresponding datasets. PLI: phase lag index; FR: frontal cortex; FT: frontotemporal regions; PA: parietal cortex; SM: somatomotor cortex; VI: visual cortex; AUC: area under the curve; HC: healthy control; SZ: schizophrenia.

Supplementary Table 1. *Effect of cost on static network measures.* The upper row contains p-values from the Friedman tests, while the lower row contains Kendall's coefficient of concordance (*W*) values. *W*=1 indicates perfect agreement among subjects. HC: healthy control; SZ: schizophrenia.

		**Connectivity Strength**		**Clustering Coefficient**		**Global Efficiency**	
		**HC**	**SZ**	**HC**	**SZ**	**HC**	**SZ**
static	*p*	<0.0001	<0.0001	<0.0001	<0.0001	<0.0001	<0.0001
	W	1	1	0.8627	0.7658	0.9985	0.9991

5.2 Mean, variance and excursions from median

Similarly to the SL-based analysis, the mean of dynamic network measures proved to be more sensitive than their static counterparts. In that, mean D, C and E was found higher in the SZ group at all cost values, except ${}^{C}\mu$ at *K*=15% (**Supplementary Figure 2**). Similarly as in the case of static FC analysis, mean values of all three network measures increased significantly with increasing the cost (**Supplementary Table 2**). Variance of D, C and E were found significantly higher in the SZ group at almost all values of K (**Supplementary Figure 2**). Increasing the cost resulted in an increase of ${}^{D}\sigma^2$ but a decrease of ${}^{C}\sigma^2$, while it has barely any effect on ${}^{E}\sigma^2$ (**Supplementary Table 2**). On the other hand, while showing a tendency similar to that of σ^2 with higher values in the SZ group, no significant difference in *EfM* was found at any value of *K*. Unsurprisingly, the AUC analysis indicated strong group-level differences, with significantly higher mean, variance and *EfM* values for all three network measures in SZ (**Supplementary Figure 3**). The AUC analysis also identified significantly higher ${}^{D}\mu$ and ${}^{D}\mu$ in the SZ group in WPLI-reconstructed dynamic networks.

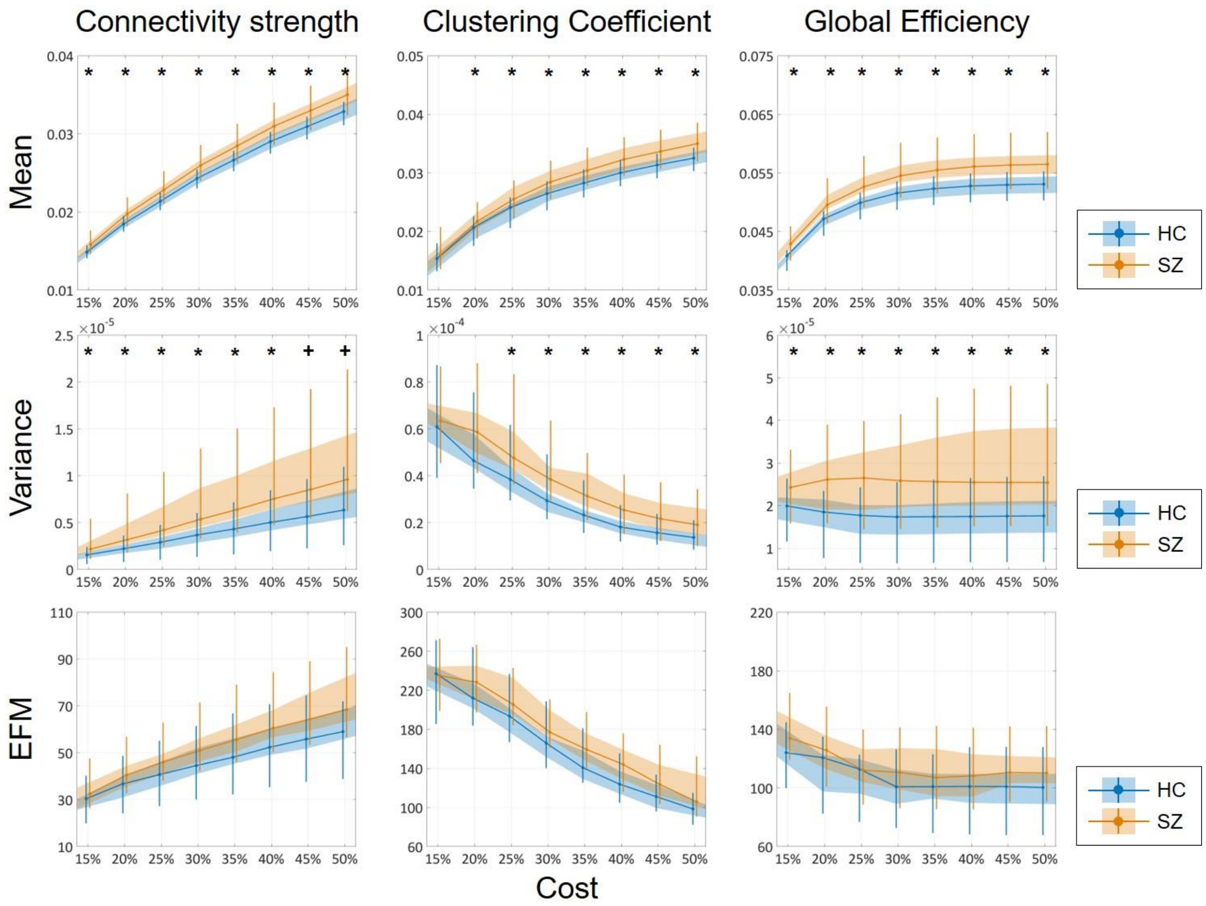

Supplementary Figure 2. *Cost-dependent results of the mean, variance and excursions from median analysis of network measures.* Mean, variance and excursions of median (EFM) values of the three network measures are plotted as functions of the cost. Black markers indicate significant group level difference (p<0.05, corrected). *****: two-sample t-test; **+**: Mann-Whitney U test; HC: healthy control; SZ: schizophrenia.

Supplementary Table 2. *Effect of cost on the mean (μ), variance (σ^2) and excursions from median (EfM) of dynamic network theoretical measures*. For each index, the upper rows contain p-values from Friedman tests, while the lower rows contain Kendall's coefficient of concordance (W) values. W=1 indicates perfect agreement among subjects. HC: healthy control; SZ: schizophrenia.

		**Connectivity Strength**		**Clustering Coefficient**		**Global Efficiency**	
		**HC**	**SZ**	**HC**	**SZ**	**HC**	**SZ**
μ	p	<0.0001	<0.0001	<0.0001	<0.0001	<0.0001	<0.0001
	W	1	1	1	1	1	1
σ^2	p	<0.0001	<0.0001	<0.0001	<0.0001	0.0027	0.0068
	W	1	1	1	0.9881	0.2225	0.1987
EfM	p	<0.0001	<0.0001	<0.0001	<0.0001	<0.0001	<0.0001
	W	0.9874	1	0.9968	0.9847	0.5401	0.3724

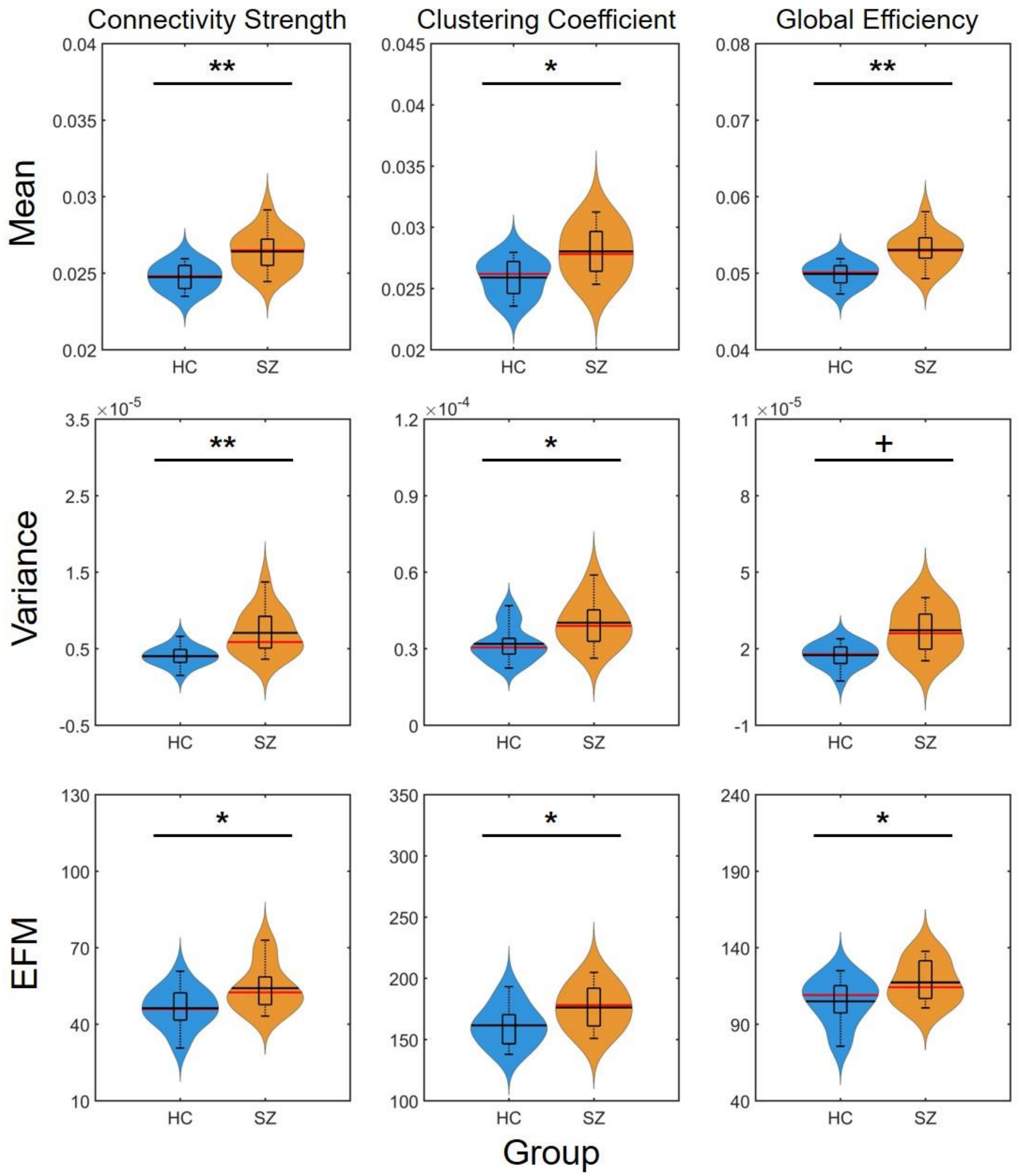

Supplementary Figure 3. *Results of the area-under-the-curve analysis regarding the mean, variance and excursions from median (EFM) of dynamic network measures*. Black markers indicate significant group level differences with one marker in case of $p<0.05$ and two markers in case of $p<0.0001$. *****: two-sample t-test; **+**: Mann-Whitney U test; HC: healthy control; SZ: schizophrenia.

5.3 Multifractal measures and entropy

When using PLI as the connectivity estimator, yet again most network measures time series (NMTS) qualified as true multifractals (**Supplementary Table 3**). The only exception is the slightly lower fraction of NMTSs that passed the phase randomization test (67.86%), however this result in itself only implicates that the observed multifractality (as verified in 100% of the time series when compared to generated monofractal signals) could not be fully explained by the presence of nonlinearity in the process. Dynamic networks reconstructed from WPLI analysis expressed similarly strong multifractality in their dynamics (**Supplementary Table 4**).

Supplementary Table 3. *Testing results for true multifractality in PLI-derived dynamic networks*. MF: multifractality; PhaseRan: phase randomization; D: connectivity strength; C: clustering coefficient; E: global efficiency; PLI: Phase Lag Index.

	Spectrum	Shuffling	True MF	PhaseRan
D	97.32%	100%	100%	67.86%
C	96.43%	100%	100%	100%
E	97.32%	100%	100%	94.64%

Supplementary Table 4. *Testing results for true multifractality in WPLI-derived dynamic networks*. MF: multifractality; PhaseRan: phase randomization; D: connectivity strength; C: clustering coefficient; E: global efficiency; WPLI: Weighted Phase Lag Index

	Spectrum	Shuffling	True MF	PhaseRan
D	96.43%	100%	100%	90.63%
C	96.88%	100%	100%	100%
E	98.21%	100%	100%	100%

Cost-dependent analysis did not reveal any significant difference in h_{max}, as well as only $^{E}FWHM$ was found significantly higher in the SZ group at K=15 and 50% (**Supplementary Figure 4**). On the other hand, ^{D}mPE and ^{C}mPE were found significantly reduced in SZ subjects for all cost values, while the same difference in ^{E}mPE was found significant only at K=50% (**Supplementary Figure 4**). Increasing K resulted in significant increase of $^{E}h_{max}$, a decrease in $^{C}h_{max}$ while it has no effect on $^{D}h_{max}$ (**Supplementary Table 5**). Similar effects were observed regarding *FWHM* with the exception that $^{E}FWHM$ decreased with increasing the cost. Comparable to results acquired from SL-based analysis, the cost had the opposite effect on *mPE* of all network measures when compared to h_{max} (**Supplementary Figure 4**, **Supplementary Table 5**). Finally, nearly identical group level differences to those of the SL-based analysis were found when comparing the AUC values of multifractal and entropy measures (**Supplementary Figure 5**).

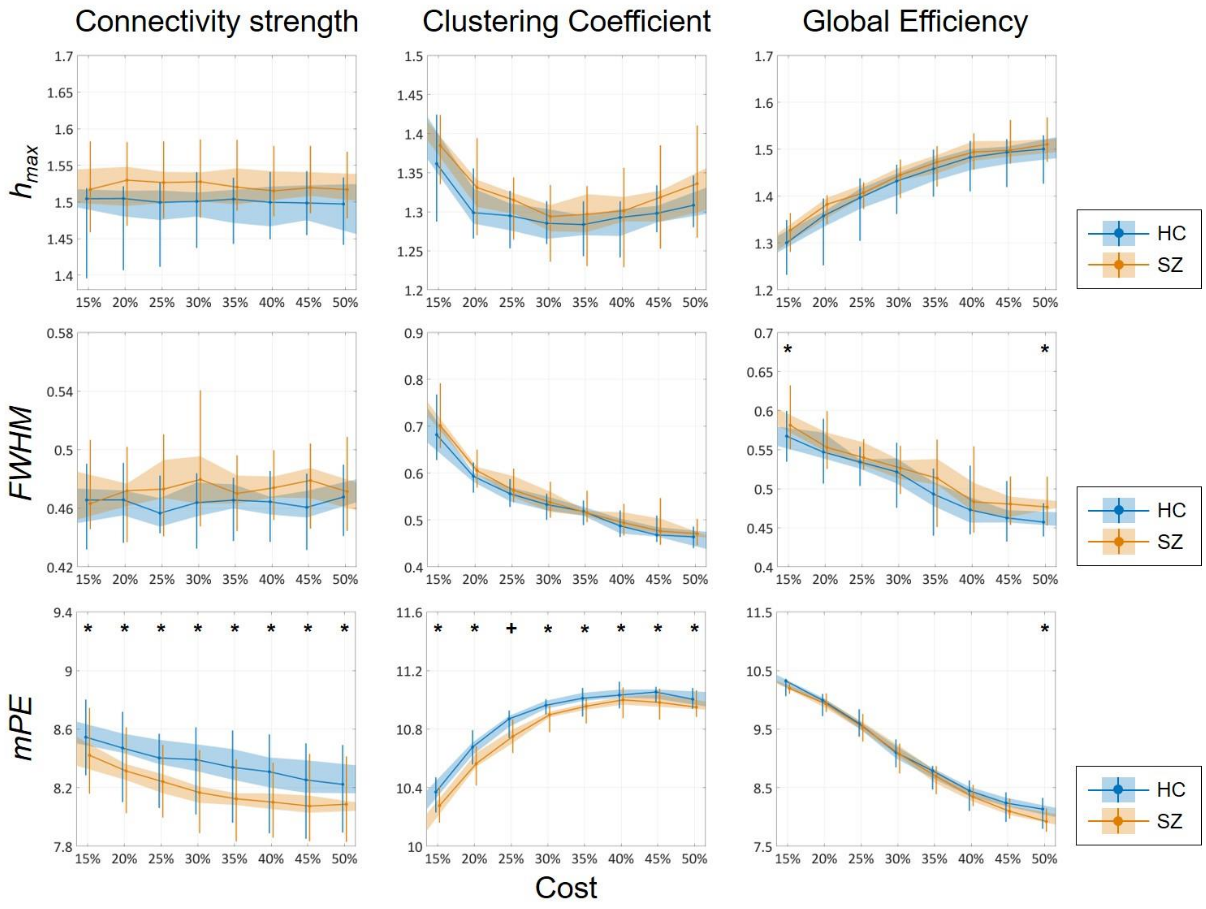

Supplementary Figure 4. *Cost-dependent results of multifractal and entropy analysis of network measures*. Multifractal measures (h_{max} and *FWHM*) and modified permutation entropy (*mPE*) of all three network measures are plotted as functions of the cost. Black markers indicate significant group level difference (p<0.05, corrected). *****: two-sample t-test; **+**: Mann-Whitney U test; HC: healthy control; SZ: schizophrenia.

Supplementary Table 5. *Effect of cost on the multifractal measures (hmax and FWHM) and modified permutation entropy (mPE) of dynamic network theoretical measures*. For each index, the upper rows contain *p*-values from Friedman tests, while the lower rows contain Kendall's coefficient of concordance (*W*) values. *W*=1 indicates perfect agreement among subjects. HC: healthy control; SZ: schizophrenia.

		**Connectivity Strength**		**Clustering Coefficient**		**Global Efficiency**	
		**HC**	**SZ**	**HC**	**SZ**	**HC**	**SZ**
h_{max}	*p*	0.8477	0.4215	<0.0001	<0.0001	<0.0001	<0.0001
	W	0.0345	0.0721	0.5189	0.5833	0.9905	0.9847
FWHM	*p*	0.3347	0.5342	<0.0001	<0.0001	0.0001	<0.0001
	W	0.0814	0.0617	0.9227	0.9269	0.8673	0.8610
mPE	*p*	0.0880	0.0385	<0.0001	<0.0001	<0.0001	<0.0001
	W	0.1266	0.1511	0.7255	0.7620	0.7925	0.8582

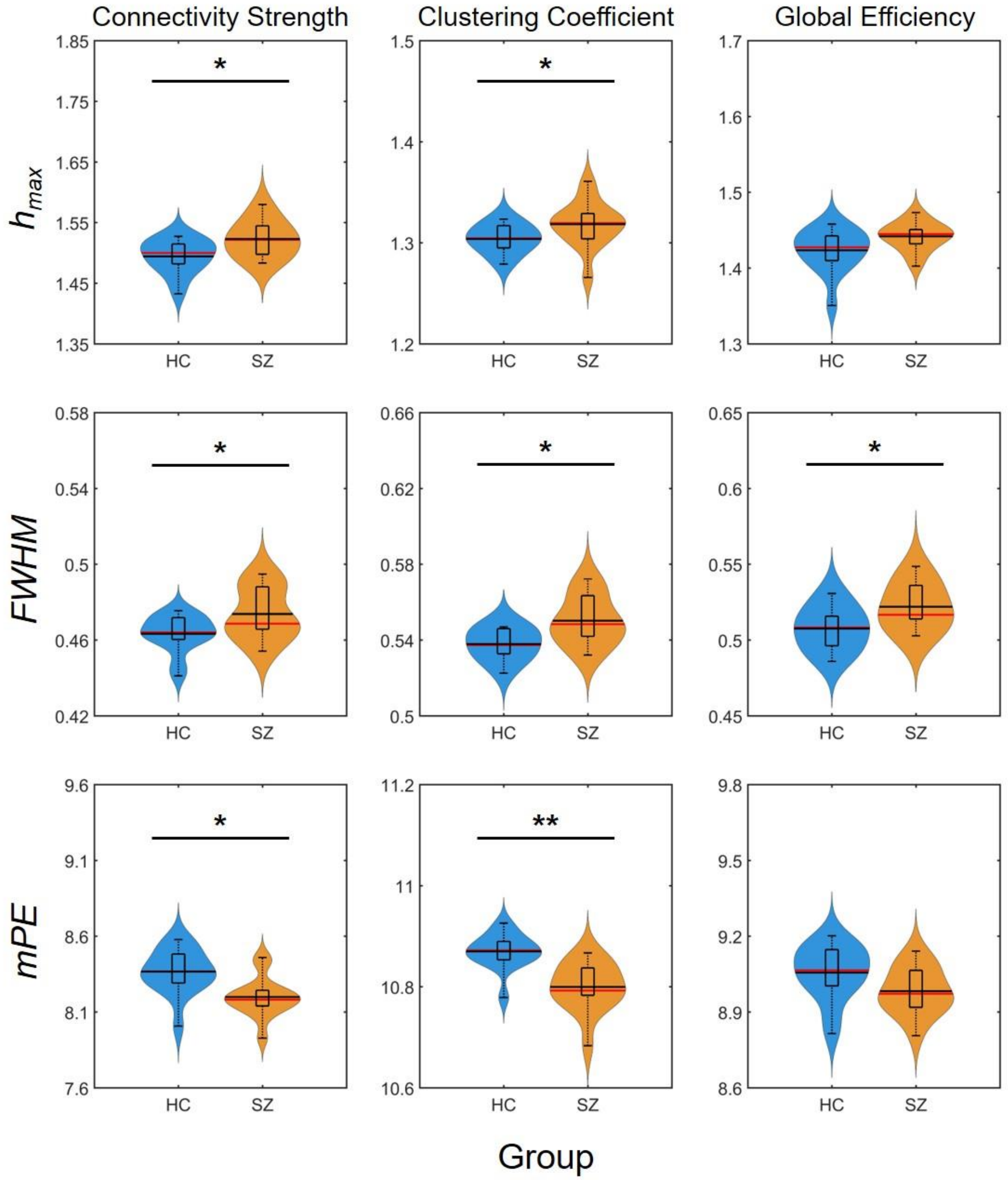

Supplementary Figure 5. *Results of the area-under-the-curve analysis regarding multifractal and entropy-related properties of dynamic network measures*. Asterisk marks significant group difference identified by two-sample t-test with one marker indicating $p<0.05$ and two markers indicating $p<0.0001$. HC: healthy control; SZ: schizophrenia. h_{max}: Hölder exponent at the peak of the multifractal spectrum; *FWHM*: full width at half maximum.

5.4 Classification and most important features

Train and test performance metrics of the classifier using AUC features derived from PLI-based analysis are shown in **Supplementary Table 5**. The classifier reached a performance comparable to that trained on SL-derived features. Surrogate datasets yielded estimates close to chance level (50%), as expected, indicating a significantly better performance of the classifier in all metrics. The cumulative Gini importance was the highest for ^{D}mPE, $^{C}\sigma^2$, $^{E}\mu$ and C_{stat} (**Supplementary Table 6**).

Supplementary Table 6. *Performance report of the random forest classifier classifiers using PLI-derived features*. ACC: accuracy; SEN: sensitivity; SPE: specificity; PPV: positive predictive value; NPV: negative predictive value; ROC-AUC: area under the receiver operator characteristic curve. CI: confidence interval; PLI: Phase Lag Index.

	**Test performance**					
	**ACC (%)**	**SEN (%)**	**SPE (%)**	**PPV (%)**	**NPV (%)**	**ROC-AUC (%)**
train	92.03	98.35	85.71	87.44	98.16	97.84
test	89.29	92.86	85.71	85.71	82.14	85.71
CI	46.93 (68.83)	47.57 (75.87)	46.29 (76.35)	33.89 (57.44)	33.25 (58.86)	45.82 (79.02)

Supplementary Table 7. *Feature importances extracted from the random forest classifier using PLI-derived indices. For each index, the network measure it was calculated from is indicated in the left superscript*. Static network measures are indicated by the subscript ’stat’ following their abbreviation. D: connectivity strength, C: clustering coefficient; E: global efficiency; stat: static; μ: mean; σ^2: variance; *EfM*: excursions from median; h_{max}: Hölder exponent at the peak of the multifractal spectrum; *FWHM*: full width at half maximum; *mPE*: modified permutation entropy; PLI: Phase Lag Index.

index	feature	importance
**1**	${}^{D}mPE$	3.7696
**2**	${}^{C}\sigma^2$	2.5557
**3**	${}^{E}\mu$	2.1993
**4**	C_{stat}	1.2970
**5**	${}^{D}\mu$	1.0521
**6**	${}^{C}h_{max}$	1.0439
**7**	${}^{E}h_{max}$	0.8477
**8**	${}^{E}EfM$	0.3316
**9**	${}^{D}h_{max}$	0.3048
**10**	${}^{C}\mu$	0.2087
**11**	${}^{C}EfM$	0.1995
**12**	D_{stat}	0.1057
**13**	${}^{E}\sigma^2$	0.0789
**14**	${}^{D}FWHM$	0.0056
**15**	${}^{C}mPE$	0.0
**16**	${}^{C}FWHM$	0.0
**17**	${}^{E}mPE$	0.0
**18**	E_{stat}	0.0
**19**	${}^{D}EfM$	0.0
**20**	${}^{D}\sigma^2$	0.0
**21**	${}^{E}FWHM$	0.0

In contrast to the lack of significant between-group differences, random forest classification was able to reach a surprisingly good performance even on AUC features derived from the WPLI-based analysis. In fact, model performance was comparable with those using SL- and PLI-derived features (**Supplementary Table 8**). The cumulative Gini importance indicated ${}^{E}h_{max}$, ${}^{C}EfM$ and D_{stat} as most important features (**Supplementary Table 9**).

Supplementary Table 8. *Performance report of the random forest classifier classifiers using WPLI-derived features*. ACC: accuracy; SEN: sensitivity; SPE: specificity; PPV: positive predictive value; NPV: negative predictive value; ROC-AUC: area under the receiver operator characteristic curve. CI: confidence interval. WPLI: Weighted Phase Lag Index.

	**Test performance**					
	**ACC (%)**	**SEN (%)**	**SPE (%)**	**PPV (%)**	**NPV (%)**	**ROC-AUC (%)**
train	96.15	93.96	98.35	98.47	94.52	99.83
test	89.29	92.86	85.71	85.71	82.14	89.29
CI	46.36<br>(70.07)	48.57<br>(79.14)	50.14<br>(79.49)	36.74<br>(60.54)	36.85<br>(61.18)	49.64<br>(79.69)

Supplementary Table 9. *Feature importances extracted from the random forest classifier using WPLI-derived indices. For each index, the network measure it was calculated from is indicated in the left superscript*. Static network measures are indicated by the subscript 'stat' following their abbreviation. D: connectivity strength, C: clustering coefficient; E: global efficiency; stat: static; μ: mean; σ^2: variance; *EfM*: excursions from median; h_{max}: Hölder exponent at the peak of the multifractal spectrum; *FWHM*: full width at half maximum; *mPE*: modified permutation entropy; WPLI: Weighted Phase Lag Index.

index	feature	importance
**1**	${}^{E}h_{max}$	2.2952
**2**	${}^{C}EfM$	1.6963
**3**	D_{stat}	1.5646
**4**	${}^{C}\mu$	1.4681
**5**	${}^{C}h_{max}$	1.2571
**6**	${}^{E}\sigma^2$	1.0985
**7**	${}^{D}\mu$	0.8020
**8**	C_{stat}	0.7334
**9**	${}^{E}\mu$	0.5931
**10**	${}^{D}\sigma^2$	0.3994
**11**	${}^{E}FWHM$	0.3552
**12**	${}^{D}EfM$	0.3287
**13**	${}^{C}mPE$	0.2834
**14**	${}^{C}FWHM$	0.2536
**15**	${}^{D}FWHM$	0.2026
**16**	${}^{D}h_{max}$	0.1969
**17**	E_{stat}	0.2895
**18**	${}^{E}mPE$	0.1408
**19**	${}^{E}EfM$	0.0761
**20**	${}^{D}mPE$	0.0455
**21**	${}^{C}\sigma^2$	0.3110

6 Classifier details

6.1 Model 1

Class: Random forest classifier

Functional connectivity estimator: Synchronization likelihood

Parameters: bootstrap=True, class_weight=None, criterion='gini', max_depth=5, max_features=3, max_leaf_nodes=None, min_impurity_decrease=0.0, min_impurity_split=None, min_samples_leaf=2, min_samples_split=2, min_weight_fraction_leaf=0.0, n_estimators=5, n_jobs=-1, oob_score=False, random_state=41904, verbose=0, warm_start=False

6.2 Model 2

Class: Random forest classifier

Functional connectivity estimator: Phase Lag Index

Parameters: bootstrap=True, class_weight=None, criterion='gini', max_depth=2, max_features=2, max_leaf_nodes=None, min_impurity_decrease=0.0, min_impurity_split=None, min_samples_leaf=2, min_samples_split=2, min_weight_fraction_leaf=0.0, n_estimators=5, n_jobs=-1, oob_score=False, random_state=845, verbose=0, warm_start=False

6.3 Model 3

Class: Random forest classifier

Functional connectivity estimator: Weighted Phase Lag Index

Parameters: bootstrap=True, class_weight=None, criterion='gini', max_depth=3, max_features='auto', max_leaf_nodes=None, min_impurity_decrease=0.0, min_impurity_split=None, min_samples_leaf=1, min_samples_split=2, min_weight_fraction_leaf=0.0, n_estimators=7, n_jobs=-1, oob_score=False, random_state=1706, verbose=0, warm_start=False

7 Definitions of classifier performance measures

During classification, a subject from the SZ group were treated as a 'positive example', while a healthy control subject as a 'negative sample'. A correctly classified positive example is referred to as a true positive (TP), while a correctly classified negative example is referred to as a true negative (TN). Conversely, a positive example incorrectly classified as negative is referred to as a false negative (FN), while a negative example incorrectly classified as positive is referred to as false positive (FP). All performance measures can be expressed using these four terms (Fawcett, 2006).

Accuracy (ACC): the proportion of correctly classified examples, expressed as

$$ACC = \frac{\sum TP + \sum TN}{\sum TP + \sum TN + \sum FP + \sum FN} \tag{3}$$

Sensitivity (SEN): also called the *true positive rate* (or *recall*), defined as the proportion of true positives to all positive examples, expressed as

$$SEN = \frac{\sum TP}{\sum TP + \sum FN} \tag{4}$$

Specificity (SPE): also called the *true negative rate*, defined as the proportion of true negatives to all negative examples, expressed as

$$SPE = \frac{\sum TN}{\sum TN + \sum FP} \tag{5}$$

Positive predictive value (PPV): also called *precision*, defined as the proportion of true positives to all examples classified as positive, expressed as:

$$PPV = \frac{\sum TP}{\sum TP + \sum FP} \tag{6}$$

Negative predictive value (NPV): the proportion of true negatives to all examples classified as negative, expressed as:

$$NPV = \frac{\sum TN}{\sum TN + \sum FN} \tag{7}$$

False positive rate (FPR): also called *fall-out*, defined as the proportion of false positives to all negative examples, and is expressed as:

$$FPR = \frac{\sum FP}{\sum FP + \sum TN} \qquad (8)$$

Receiver operator characteristic (ROC): the ROC curve is acquired by plotting the sensitivity (true positive rate) against the false positive rate at various threshold values and is most frequently characterized by its area under the curve (AUC). In case of perfect classification it takes its maximal possible value of 1, while in case of a dummy classifier and a balanced sample (where the proportion of positive and negative examples is 50-50%) it takes a value close to its expected value of 0.5.

8 References

Fawcett T 2006. An introduction to ROC analysis.*Pattern Recognition Letters* **27**(8)**,** 861-74. doi:10.1016/j.patrec.2005.10.010

Hardmeier M, Hatz F, Bousleiman H, Schindler C, Stam C J and Fuhr P 2014. Reproducibility of Functional Connectivity and Graph Measures Based on the Phase Lag Index (PLI) and Weighted Phase Lag Index (wPLI) Derived from High Resolution EEG.*PLoS One* **9**(10). doi:ARTN e108648 10.1371/journal.pone.0108648

Rosenblum M G, Pikovsky A S and Kurths J 1996. Phase synchronization of chaotic oscillators.*Physical Review Letters* **76**(11)**,** 1804-7. doi:DOI 10.1103/PhysRevLett.76.1804

Stam C J, Nolte G and Daffertshofer A 2007. Phase lag index: Assessment of functional connectivity from multi channel EEG and MEG with diminished bias from common sources.*Human brain mapping* **28**(11)**,** 1178-93. doi:10.1002/hbm.20346

Stam C J and van Dijk B W 2002. Synchronization likelihood: an unbiased measure of generalized synchronization in multivariate data sets.*Physica D-Nonlinear Phenomena* **163**(3-4)**,** 236-51. doi:10.1016/S0167-2789(01)00386-4

Vinck M, Oostenveld R, van Wingerden M, Battaglia F and Pennartz C M A 2011. An improved index of phase-synchronization for electrophysiological data in the presence of volume-conduction, noise and sample-size bias.*Neuroimage* **55**(4)**,** 1548-65. doi:10.1016/j.neuroimage.2011.01.055
